# Tau Cleavage Contributes to Cognitive Dysfunction in Strepto-Zotocin-Induced Sporadic Alzheimer’s Disease (sAD) Mouse Model

**DOI:** 10.3390/ijms222212158

**Published:** 2021-11-10

**Authors:** Valentina Latina, Giacomo Giacovazzo, Pietro Calissano, Anna Atlante, Federico La Regina, Francesca Malerba, Marco Dell’Aquila, Egidio Stigliano, Bijorn Omar Balzamino, Alessandra Micera, Roberto Coccurello, Giuseppina Amadoro

**Affiliations:** 1 European Brain Research Institute (EBRI), Viale Regina Elena 295, 00161 Rome, Italy; v.latina@ebri.it (V.L.); pietro.calissano@gmail.com (P.C.); f.laregina@ebri.it (F.L.R.); f.malerba@ebri.it (F.M.); 2 IRCSS Santa Lucia Foundation, Via Fosso del Fiorano 64-65, 00143 Rome, Italy; giacomogiacovazzo@virgilio.it; 3 Institute of Biomembranes, Bioenergetics and Molecular Biotechnologies (IBIOM)-CNR, Via Amendola 122/O, 70126 Bari, Italy; a.atlante@ibiom.cnr.it; 4 Area of Pathology, Department of Woman and Child Health and Public Health, Fondazione Policlinico Universitario A. Gemelli IRCCS, Istituto di Anatomia Patologica, Università Cattolica del Sacro Cuore, Largo Francesco Vito, 1, 00168 Rome, Italy; mzrk07@gmail.com (M.D.); egidio.stigliano@policlinicogemelli.it (E.S.); 5 Research Laboratories in Ophthalmology, IRCCS-Fondazione Bietti, Via Santo Stefano Rotondo, 6I, 00184 Rome, Italy; bijorn.balzamino@fondazionebietti.it (B.O.B.); alessandra.micera@fondazionebietti.it (A.M.); 6 Institute for Complex System (ISC)-CNR, Via dei Taurini 19, 00185 Rome, Italy; 7 Institute of Translational Pharmacology (IFT)-CNR, Via Fosso del Cavaliere 100, 00133 Rome, Italy

**Keywords:** tau cleavage, non-transgenic Alzheimer’s Disease mouse model, streptozotocin (STZ), immunotherapy, cognitive/memory impairment, neuroinflammation, oxidative stress

## Abstract

Tau cleavage plays a crucial role in the onset and progression of Alzheimer’s Disease (AD), a widespread neurodegenerative disease whose incidence is expected to increase in the next years. While genetic and familial forms of AD (fAD) occurring early in life represent less than 1%, the sporadic and late-onset ones (sAD) are the most common, with ageing being an important risk factor. Intracerebroventricular (ICV) infusion of streptozotocin (STZ)—a compound used in the systemic induction of diabetes due to its ability to damage the pancreatic β cells and to induce insulin resistance—mimics in rodents several behavioral, molecular and histopathological hallmarks of sAD, including memory/learning disturbance, amyloid-β (Aβ) accumulation, tau hyperphosphorylation, oxidative stress and brain glucose hypometabolism. We have demonstrated that pathological truncation of tau at its N-terminal domain occurs into hippocampi from two well-established transgenic lines of fAD animal models, such as Tg2576 and 3xTg mice, and that it’s in vivo neutralization via intravenous (i.v.) administration of the cleavage-specific anti-tau 12A12 monoclonal antibody (mAb) is strongly neuroprotective. Here, we report the therapeutic efficacy of 12A12mAb in STZ-infused mice after 14 days (short-term immunization, STIR) and 21 days (long-term immunization regimen, LTIR) of i.v. delivery. A virtually complete recovery was detected after three weeks of 12A12mAb immunization in both novel object recognition test (NORT) and object place recognition task (OPRT). Consistently, three weeks of this immunization regimen relieved in hippocampi from ICV-STZ mice the AD-like up-regulation of amyloid precursor protein (APP), the tau hyperphosphorylation and neuroinflammation, likely due to modulation of the PI3K/AKT/GSK3-β axis and the AMP-activated protein kinase (AMPK) activities. Cerebral oxidative stress, mitochondrial impairment, synaptic and histological alterations occurring in STZ-infused mice were also strongly attenuated by 12A12mAb delivery. These results further strengthen the causal role of N-terminal tau cleavage in AD pathogenesis and indicate that its specific neutralization by non-invasive administration of 12A12mAb can be a therapeutic option for both fAD and sAD patients, as well as for those showing type 2 diabetes as a comorbidity.

## 1. Introduction

Alzheimer’s Disease (AD), the leading cause of dementia, involves genetic, environmental and metabolic factors, including alterations in brain insulin signaling and glucose metabolism, which occur several years before the clinical symptoms become evident [1,2,3]. Amyloid-based models rely on the use of transgenic animals for the expression of mutations linked to familial forms of AD (fAD) in the causative genes for amyloid precursor protein (APP), presenilin 1 (PS1) and presenilin 2 (PS2). Nevertheless, although these in vivo paradigms are valuable tools to study the molecular mechanisms underlying the AD etiopathogenesis, they do not replicate all aspects of disease’s neuropathology seen in human patients and are quite dissimilar from the most common sporadic forms (sAD) in which no APP overexpression and/or mutation have been reported up to now. From a translational point of view, non-transgenic animal models represent a complementary and useful alternative for preclinical studies of drug discovery and intervention strategies by providing a more holistic, pathological picture of this complex and heterogeneous disorder with multiple etiological conditions [4].

A growing body of epidemiological evidence has shown that alterations in the insulin-signaling pathway linked with type-2 diabetes (insulin resistance) significantly increase the risk of developing dementia and/or of progression from mild cognitive impairment (MCI) to full blown AD [2,5,6,7]. Numerous studies have reported that the human diabetic brain exhibits neuroinflammation, mitochondrial dysfunction, oxidative stress resulting in hippocampal vulnerability and synaptic damage [8,9,10]. In this context, intracerebroventricular (ICV) infusion(s) of streptozotocin (STZ), a diabetogenic compound inducing in a laboratory animal a systemic resistance to insulin, can mimic some pathological aspects observed in brain of sAD subjects. Indeed, adult rodents that underwent STZ infusion develop cerebral accumulation of Aβ [11], increased tau phosphorylation and aggregation [12,13], oxidative stress as revealed by increased levels of malondialdehyde and decreased levels of glutathione [14,15,16], changes in insulin signaling [17], dysmetabolism of glucose and energy production [18,19,20], down-regulation of ChAT activity [21], gliosis [12,22,23], caspase(s) activation and apoptosis [24], alteration in synaptic proteins and long-term cognitive deficits [20,25,26,27,28]. The insulin/phosphoinositide 3-kinase/protein kinase B/glycogen synthase kinase-3β (insulin/PI3K/AKT/GSK3-β) pathway, which regulates glucose metabolism in the brain [20,29,30], is down-regulated in ICV-infused STZ mice along with inactivation of AMP-activated protein kinase (AMPK) signaling cascade, a kinase acting as master sensor of energy balance in mammalian cells [31]. Thus, it is widely accepted that ICV-STZ delivery produces brain cerebral glucose deregulation and insulin resistance, which eventually lead to the development of Aβ and tau pathology resembling those detected in sAD cases [17,32]. ICV-STZ administration is routinely used as an experimental, non-transgenic metabolic animal model for the study of sporadic AD (sAD) [32,33,34]. In addition, the induction of insulin-resistant brain state following ICV-STZ further exacerbates the biochemical, immunohistochemical and behavioral hallmarks in two different models of hereditary AD, such as Tg2576 (APPKM670/671NL Swedish mutation) [35] and 3xTg (APP Swedish, MAPT P301L, and PSEN1 M146V) [36] mice. 

Mounting evidence suggests that, not only the hyper-phosphorylation, but also the tau cleavage plays a crucial role in the development and progression of AD [37,38]. In this context, we have recently demonstrated that: (i) the neurotoxic 20–22 kDa peptide deriving from the pathological truncation of tau at its N-terminal domain (aka NH_2_htau) accumulates into hippocampi from two well-established transgenic lines of fAD animal models, such as Tg2576 and 3xTg mice; (ii) its neutralization, following intravenous (i.v.) injection of the cleavage-specific anti-tau 12A12mAb, improves several relevant neuropathological and behavioral hallmarks of symptomatic (6-month-old) animals [1]; (iii) pathological N-truncation also participates in retinas and vitreous body deterioration associated with AD phenotype of Tg2576 mice and its deleterious effects are successfully antagonized by 12A12mAb immunization [39].

**Figure 1:**
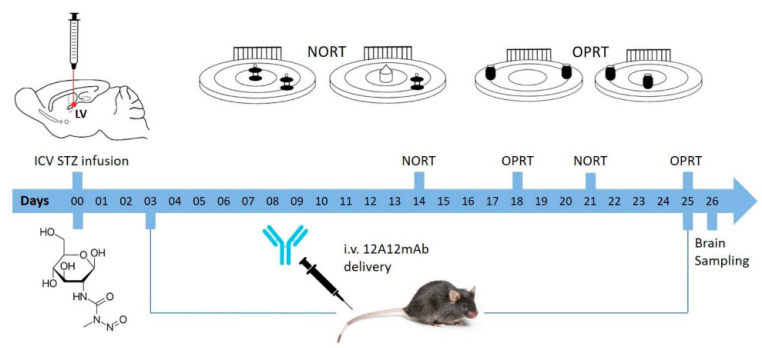
Illustration of experimental design. Establishment of streptozotocin (STZ)-induced sporadic Alzheimer’s Disease (sAD) mouse model, immunization regimen and experimental procedures are shown. The mice were divided into four groups: STZ 0 plus vehicle; STZ 0 plus mAb; STZ 3 plus vehicle; STZ 3 plus mAb and housed in groups. The dose of 12A12mAb administered to mice in this study was calculated according to [1,39] and administered by intravenous injection into the lateral caudal vein on two alternated days up to 3 weeks.

To further explore the therapeutic efficacy of 12A12mAb prior to advance to clinical trials, we evaluated the effects of its systemic administration on ICV-STZ mice phenotype. This well-established non-transgenic AD animal model is considered a gold standard to advance the preclinical development of potential drug and/or antibody candidates aimed at treating patients which are for the large part (about 90%) affected from the sporadic non-familial type. To address this purpose, we surgically infused the STZ in 6-month-old male C57BL/6J mice, which was given bilaterally into cerebral ventricles in single bolus (3 mg/Kg in 3 μL/side) alone or with 12A12mAb. This antibody was systemically delivered into lateral caudal vein on alternate days up to three weeks, as previously reported [1,39]. Animals were tested for their cognitive performance after 14 (short-term immunization regimen) and 21 (long-term immunization regimen) days [12,40]. Two days after the last immunization, animals were sacrificed for biochemical, histological and metabolic evaluations on their isolated hippocampi and cortices (Figure 1). Interestingly, we found that the most relevant AD-like cognitive, neurochemical and anatomopathological abnormalities classically detected in ICV-STZ mice under our experimental conditions were significantly improved by neutralization of the endogenously generated NH_2_htau following its removal via 12A12mAb administration. These findings provide new insights into the pivotal role of N-terminal tau cleavage in AD pathogenesis and indicate that it is in vivo targeted inhibition can be a novel therapeutic agent for the treatment of both familial [1,39] and sporadic forms of AD. 

## 2. Results

### 2.1. STZ-Induced sAD-Like Deficits of Memory Function Are Rescued by 12A12mAb Immunization

The main goals of this study were to test whether: (i) N-terminal tau cleavage occurred in ICV-STZ preclinical model of sAD; (ii) 12A12mAb delivery exerted an in vivo beneficial action on the cognitive and neurochemical and neuropathological hallmarks associated with animals’ AD phenotype.

**Figure 2:**
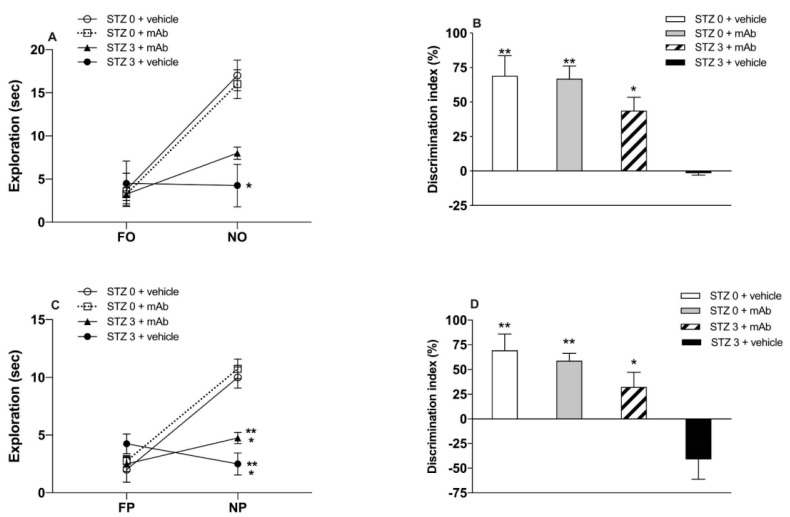
Novel object recognition test (NORT) and object place recognition task (OPRT) cognitive scores of ICV-STZ mice after short-term immunization regimen with 12A12mAb. (**A**) The panel depicts the level of exploration (sec) during the NORT test phase. Unlike naïve ICV-STZ mice (STZ 3 plus vehicle), both groups of non-STZ-infused mice (STZ 0 plus vehicle and STZ 0 plus mAb) significantly directed their exploratory activity towards the novel object (NO), irrespective of mAb treatment (* *p* < 0.05). ICV-STZ mice that underwent i.v. delivery of 12A12mAb (STZ 3 plus mAb) showed a trend of increase of NO exploration which, however, was not statistically different from that of their non-immunized counterparts (STZ 3 plus vehicle vs. STZ 3 plus mAb, n.s.). (**B**) The panel depicts the level of DI during the NORT test phase, showing that naïve ICV-STZ mice (STZ 3 plus vehicle) were severely cognitively impaired, being unable to discriminate between the familial (FO) and NO objects (i.e., negative DI index). On the contrary, both groups of non-STZ mice (STZ 0 plus vehicle and STZ 0 plus mAb) showed significantly higher DI (** *p* < 0.01) in comparison with naïve ICV-STZ mice (STZ 3 plus vehicle). An intermediate level of DI was showed by ICV-STZ mice that underwent immunization (STZ 3 plus mAb vs. STZ 3 plus vehicle, * *p* < 0.05). (**C**) The panel depicts the level of exploration (sec) during the OPRT test phase. Naïve ICV-STZ mice (STZ 3 plus vehicle) did not explore the NP, while both groups of non-STZ-infused mice significantly directed their exploratory activity towards the NP, irrespective of mAb treatment (STZ 3 plus vehicle vs STZ 0 plus mAb, ** *p* < 0.01; STZ 3 plus vehicle vs. STZ 0 plus vehicle, * *p* < 0.05). ICV-STZ mice that underwent immunization (STZ 3 plus mAb) showed an increase of NP exploration which was, however, still lower than that of non-STZ mice (STZ 3 plus mAb vs. STZ 0 plus mAb, ** *p* < 0.01; STZ 3 plus mAb vs. STZ 0 plus vehicle, * *p* < 0.05). (**D**) The panel depicts the level of DI during the OPRT test phase, showing the complete lack of discrimination (i.e., negative DI index) exhibited by naïve ICV-STZ mice (STZ 3 plus vehicle). In contrast, both groups of non-STZ mice (STZ 0 plus vehicle and STZ 0 plus mAb) displayed significantly higher DI (** *p* < 0.01) in comparison with naïve ICV-STZ mice. ICV-STZ mice that underwent i.v. delivery of 12A12mAb showed an intermediate level of DI as compared to non-immunized counterpart (STZ 3 plus mAb vs. STZ 3 plus vehicle, * *p* < 0.05).

**Figure 3:**
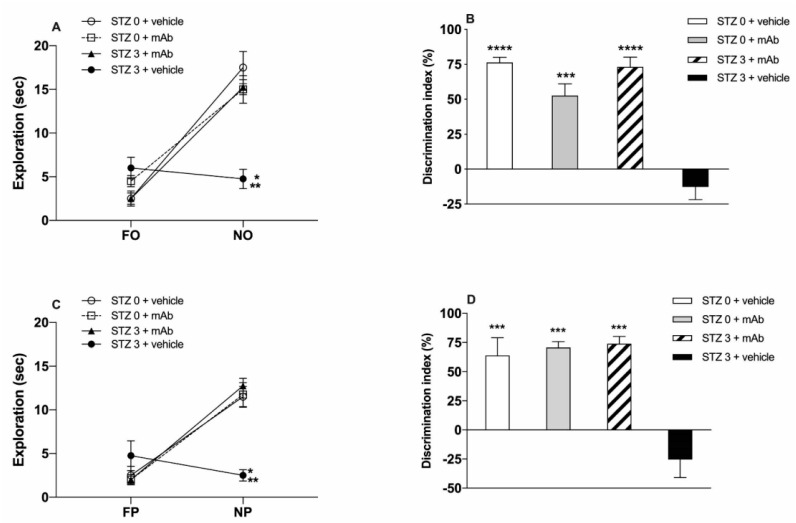
NORT and OPRT cognitive scores of ICV-STZ mice after long-term immunization regimen with 12A12mAb. (**A**) The panel depicts the level of exploration (sec) during the NORT test phase. Unlike naïve ICV-STZ mice (STZ 3 plus vehicle), both non-STZ-infused mice (STZ 0 plus vehicle and STZ 0 plus mAb) as well as ICV-STZ mice that underwent immunization with 12A12mAb (STZ 3 plus mAb) significantly oriented their exploratory activity towards the NO (STZ 3 plus vehicle vs. STZ 0 plus mAb and STZ 0 plus vehicle, ** *p* < 0.01; STZ 3 plus vehicle vs. STZ 3 plus mAb, * *p* < 0.05). (**B**) The panel depicts the level of DI during the NORT test phase, showing that non-immunized ICV-STZ mice (STZ 3 plus vehicle) were severely cognitively impaired, being unable to discriminate between the FO and NO objects (i.e., negative DI index). On the contrary, both STZ 0 plus vehicle and STZ 3 plus mAb groups, and also STZ 0 plus mAb mice showed significantly higher DI in comparison with non-immunized ICV-STZ mice (STZ 0 plus vehicle and STZ 3 plus mAb vs STZ 3 plus vehicle, **** *p* < 0.0001; STZ 0 plus mAb vs STZ 3 plus vehicle, *** *p* < 0.0005). (**C**) The panel depicts the level of exploration (sec) during the OPRT test phase. Naïve ICV-STZ mice (STZ 3 plus vehicle) did not explore the novel place (NP). On the contrary, both non-STZ mice (STZ 0 plus vehicle and STZ 0 plus mAb) and ICV-STZ-infused mice that underwent immunization (STZ 3 plus mAb) significantly oriented their exploratory activity towards the NP (STZ 3 plus vehicle vs. STZ 0 plus mAb and STZ 0 plus vehicle, * *p* < 0.05; STZ 3 plus vehicle vs. STZ 3 plus mAb, ** *p* < 0.01). (**D**) The panel depicts the level of DI during the OPRT test phase, showing the complete lack of discrimination (i.e., negative DI index) of naïve ICV-STZ mice (STZ 3 plus vehicle). On the contrary, both groups of non-STZ mice (STZ 0 plus vehicle and STZ 0 plus mAb) and ICV-STZ immunized mice (STZ 3 plus mAb) showed significantly higher DI (*** *p* < 0.0005) in comparison with naïve counterpart (STZ 3 plus vehicle).

STZ-induced sAD mice underwent the novel object recognition test (NORT) (Figure 2A,B and Figure 3A,B) and object place recognition task (OPRT) (Figure 2C,D and Figure 3C,D) to evaluate the impact of the 12A12mAb immunization on their cognitive performance. ICV-STZ sAD mice were either immunized up to three weeks with injections of 12A12mAb delivered on two alternate days to the lateral vein of the tail (i.e., STZ 3 plus mAb group) or treated with 12A12mAb vehicle following the same schedule and route of administration (i.e., STZ 3 plus vehicle group). Age-matched animals infused under the same experimental conditions with either STZ vehicle and 12A12mAb vehicle (i.e., STZ 0 plus vehicle group) or STZ vehicle and 12A12mAb (i.e., STZ 0 plus mAb group) were included as negative controls. The assessment of cognitive deficits was scheduled in two separate time points, as follows: (i) 12–13 days after STZ infusion (short-term immunization regimen, STIR), mice were evaluated in the NORT (Figure 2A,B), and 14–15 days after STZ infusion the same animals were assessed in the OPRT (Figure 2C,D); (ii) 21–22 days after STZ infusion (long-term immunization regimen, LTIR), mice were evaluated in the NORT (Figure 3A,B) and 23–24 days after STZ infusion the same animals were assessed in the OPRT (Figure 3C,D). No differences in the time spent exploring the two identical objects during the sample phase were found among animals’ cohorts (data not shown), while the short-term memory performances during the test phase are showed via the absolute time (sec) of exploration of novel object (i.e., NORT) or novel place (i.e., OPRT), as well as via the discrimination index (DI). 

Results after STIR showed that, in the NORT task (Figure 2A,B), STZ-infused mice (STZ 3 plus vehicle) spent a lower amount of time in the exploration of the novel object (NO) when compared with both groups of non-STZ-infused mice (STZ 0 plus vehicle and STZ 0 plus mAb), irrespective of the antibody administration. Two-way ANOVA revealed a treatment effect (F _(3,24)_ = 5.597, * *p* < 0.01), a NO effect (F _(1,24)_ = 37.46, *** *p* < 0.0001) and a treatment x NO interaction (F _(3,24)_ = 6.883, ** *p* < 0.0005). Post-hoc analysis further revealed that STZ-infused mice that did not receive the 12A12mAb immunization (STZ 3 plus vehicle) were impaired in their ability to significantly explore the NO (Figure 2A), thus indicating that ICV-STZ infusion per se induced a net impairment of recognition memory in agreement with previous data [41]. Moreover, these data showed that both groups of non-STZ-infused mice explored the NO significantly more than STZ-infused mice (STZ 0 plus vehicle and STZ 0 plus mAb vs. STZ 3 plus vehicle), regardless of 12A12mAb immunization (Figure 2A). Concerning the analysis of DI, the one-way ANOVA confirmed the existence of a significant treatment effect (F _(3,12)_ = 10.87, *** *p* < 0.001), and the post-hoc analysis further disclosed that STZ-infused and 12A12mAb-immunized mice (STZ 3 plus mAb group) showed a DI higher than STZ-infused but not 12A12mAb-immunized mice (Figure 2B). It should, however, be noted that the STIR schedule of 12A12mAb immunization did not produce a complete recovery of the impairment in the discrimination of the NO, as evidenced by the higher DI showed by both groups of non-immunized mice (Figure 2B). 

The analysis of cognitive performance (i.e., spatial memory) in the OPRT after ICV-STZ infusion showed that non-immunized mice (STZ 3 plus vehicle) did not react to the novel spatial location of the object (novel placement, NP) by spending significantly less time in the NP exploration, as compared to both non-STZ-infused mice (STZ 0 plus vehicle and STZ 0 plus mAb), irrespective of the 12A12mAb immunization (Figure 2C). The two-way ANOVA analysis showed a significant treatment effect (F _(3,24)_ = 8.714), a significant NP effect (F _(1,24)_ = 51.86, **** *p* < 0.0001) as well as significant treatment x NP interaction (F _(3,24)_ = 17.29, **** *p* < 0.0001). Interestingly, STZ-infused, 12A12mAb-immunized mice (STZ 3 plus mAb) displayed the tendency to a higher exploration of NP than not-immunized mice (STZ 3 plus vehicle), although this did not correspond to a statistically significant difference (Figure 2C). By contrast, the analysis of DI showed a significant treatment effect (F _(3,12)_ = 10.40, ** *p* < 0.01), and the post-hoc comparison further revealed that STZ-infused, 12A12mAb-immunized mice significantly discriminated the NP as compared to not-immunized mice (STZ 3 plus vehicle), although the DI was lower than that exhibited by both groups of non-STZ-infused mice (Figure 2D). Hence, these data further confirm the in vivo neuroprotective action of 12A12mAb against the impairment of not only recognition memory but also spatial memory scores. Nevertheless, it is worth noting that in both NORT and OPRT performances the level of exploration of novelty (NO and NP) in STZ 3 plus mAb mice was significantly higher than that of non-immunized animals (STZ 3 plus vehicle), but still lower than that of both non-STZ-infused groups (STZ 0 plus vehicle and STZ 0 plus mAb). Thus, as for the NORT performance, the STIR schedule of 12A12mAb immunization did not produce a full recovery of the impairment in the discrimination. On the other hand, under LTIR, the delivery of 12A12mAb exerted a more pronounced and a sustained recovery effect on STZ-induced short-term memory impairment. Two-way ANOVA revealed a treatment effect (F _(3,24)_ = 6.677, ** *p* < 0.01), NO effect (F _(1,24)_ = 124.9, **** *p* < 0.0001) and treatment x FO interaction (F _(3,24)_ = 19.12, **** *p* < 0.0001). The post-hoc analysis further disclosed that, in the NORT test, the level of exploration of NO in ICV-STZ mice receiving the antibody was not only significantly higher than that of sham-immunized counterparts (STZ 3 plus vehicle vs. STZ 3 plus mAb, *p* < 0.05) but also reached approximately that of both non-STZ-infused mice (STZ 0 plus vehicle and STZ 0 plus mAb) (Figure 3A). Moreover, one-way ANOVA of the DI showed a significant treatment effect (F _(3,12)_ = 31.83, **** *p* < 0.0001), and the post-hoc analysis showed that the only group of animals impaired in the NORT performance was that of STZ-infused mice (STZ 3 plus vehicle) as compared to all the other groups, including the group of STZ-infused but immunized animals (STZ 3 plus vehicle vs. STZ 3 plus mAb) (Figure 3B). 

Similarly, STZ-infused, but non-immunized animals (STZ 3 plus vehicle) assessed in the OPRT displayed a lack of exploration of the NP in sharp contrast to the level of exploration exhibited by all the other experimental groups (Figure 3C). Two-way ANOVA revealed a significant treatment effect (F _(3,24)_ = 5.43, ** *p* < 0.01), NP effect (F _(1,24)_ = 83.08, **** *p* < 0.0001) and significant treatment x NP interaction (F _(3,24)_ = 16.57, **** *p* < 0.0001). In particular, the post-hoc analysis disclosed that both groups of STZ naïve animals (STZ 0 plus vehicle and STZ 0 plus mAb) as well as STZ-infused and immunized mice (STZ 3 plus mAb) significantly explored the NP (Figure 3C). Moreover, one-way ANOVA of the DI showed a significant treatment effect (F _(3,12)_ = 16.98, **** *p* < 0.0001), and the post-hoc analysis further confirmed that 12A12mAb immunization induced a full recovery of the impairment of spatial memory induced by the STZ infusion (STZ 3 plus mAb vs. STZ 3 plus vehicle, *p* < 0.0005; Figure 3D), and the immunization did not affect the DI of non-STZ-infused mice (Figure 3D).

### 2.2. STZ-Induced sAD-like Pathological Hallmarks Are Mitigated by 12A12mAb Treatment

**Figure 4:**
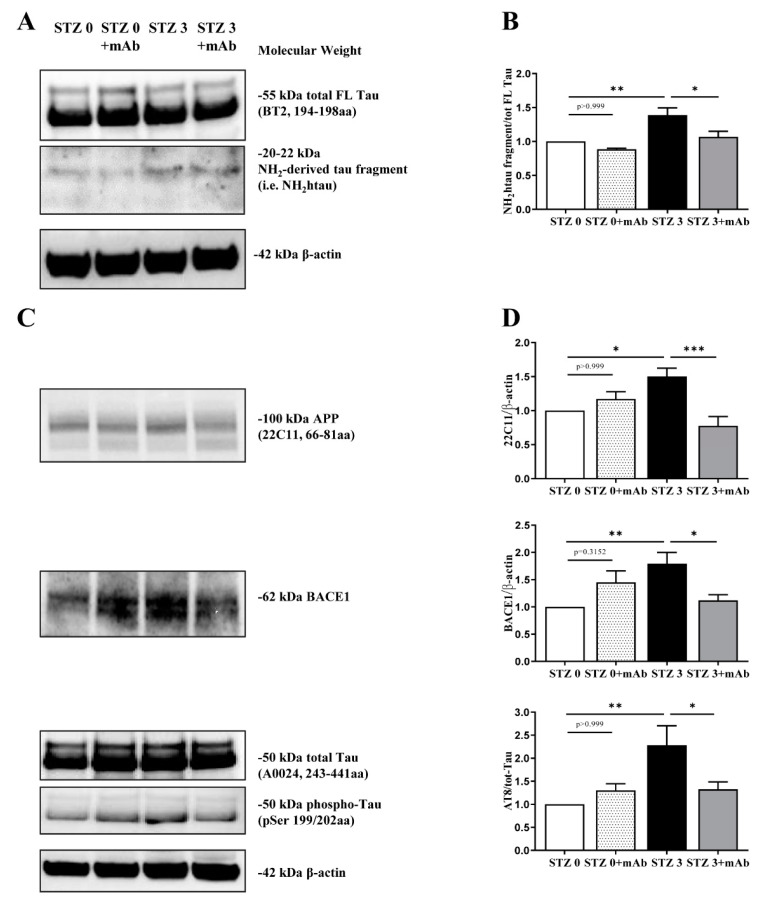
The 12A12mAb-mediated neutralization of the N-terminal tau cleavage in brains from ICV-STZ mice is associated with reduction in AD-linked, pathologically relevant protein hallmarks. (**A**–**C**) Hippocampal homogenates from animals of four experimental groups (STZ 0 plus vehicle; STZ 0 plus mAb; STZ 3 plus vehicle; STZ 3 plus mAb) were analyzed by Western blotting by developing with antibodies reported alongside the blot; (**B**–**D**) semi-quantitative densitometry of the intensity signals of bands was carried out following normalization with β-actin level used as loading control. Arrows on the right side indicate the molecular weight (kDa) of bands calculated from migration of standard proteins. Values are from at least three independent experiments and statistically significant differences were calculated by one-way ANOVA followed by Bonferroni’s post-hoc test for multiple comparison among more than two groups. *p* < 0.05 was accepted as statistically significant (* *p* < 0.05; ** *p* < 0.01; *** *p* < 0.0005).

In view of these preliminary behavioral results, in order to correlate the cognitive alterations with molecular changes, we assessed the neurochemical effects of immunization protocol only at day 26 after STZ infusion, in agreement with previously published studies in this sAD animal model [12,36,40]. Western blotting SDS-PAGE analyses were carried out on hippocampal protein extracts from four experimental groups (STZ 0 plus vehicle; STZ 0 plus mAb; STZ 3 plus vehicle; STZ 3 plus mAb) to assess the presence of the neurotoxic N-terminal tau peptide (known as NH_2_htau) into their hippocampi and its response to 12A12mAb treatment. As shown in Figure 4A,B, BT2, the pan-tau antibody directed against the 194–198 amino acids of full-length (FL) protein, evidenced that a low but sizeable amount of the NH_2_htau fragment with expected 20–22 kDa molecular weight (MW) was specifically generated into the hippocampi of STZ-infused mice (one-way ANOVA followed by Bonferroni’s post-hoc test, STZ 3 plus vehicle vs. STZ 0 plus vehicle, ** *p* < 0.01). These results are similar to those we found in the hippocampi from AD patients [38], indicating that the N-terminal tau truncation with generation of toxic fragment(s) is a common pathogenic event of both fAD [1,38] and sAD forms. In addition, these findings extended previous results [42] showing that cleavage of tau is increased in hippocampus of ICV-STZ mice, as previously reported in this sAD animal model for another post-translational modification, such as hyperphosphorylation at multiple disease-associated sites [12]. Furthermore, immunization following 12A12mAb delivery significantly reduced the endogenous level of the NH_2_htau in STZ 3 plus mAb group, as compared with the STZ 3 plus vehicle controls (one-way ANOVA followed by Bonferroni’s post-hoc test, STZ 3 plus vehicle vs. STZ 3 plus mAb, * *p* < 0.05). Antibody treatment is ineffective per se in altering the intracellular amount of the NH_2_htau when STZ 0 plus vehicle mice were compared with their antibody-treated counterpart (STZ 0 plus vehicle vs. STZ 0 plus mAb, *p* > 0.999). Importantly, no changes in the steady-state expression level of total FL tau were contextually detected in any group (*p* > 0.999), in consistency with tau cleavage-specificity of 12A12mAb, which selectively binds the neurotoxic NH_2_htau truncated specie(s) in vivo [1,43]. 

After that, we evaluated whether the most prominent sAD-like alterations in the ICV-STZ mice—such as the up-regulation of intracellular expression of the amyloid precursor protein (APP), BACE-1 (β-site of the amyloid precursor protein cleaving enzyme) a proteinase generating neurotoxic β-amyloid (Aβ) and tau hyper-phosphorylation (phospho-tau) at Ser199/Thr202 sites (AT8 epitope)—were inhibited by i.v. 12A12mAb delivery. As shown in Figure 4C,D, and in line with previous findings [12,17], the increase in the immunoreactivity levels of APP, BACE-1 and phospho-tau detected in the STZ 3 plus vehicle group (STZ 3 plus vehicle vs. STZ 0 plus vehicle, * *p* < 0.05; ** *p* < 0.01) confirmed that derangement of insulin signaling induced by ICV-STZ infusion led to the activation of amyloidogenic pathway with Aβaccumulation and tau pathology, just as reported to occur in human sAD brain [32]. Notably, 12A12mAb delivery strongly decreased the APP, BACE-1 and phospho-tau levels in STZ 3 plus mAb group when compared with its non-immunized counterpart (STZ 3 plus vehicle vs. STZ 3 plus mAb, * *p* < 0.05; *** *p* < 0.0005) up to physiological baseline. Any apparent change in intensity signal between STZ 0 plus vehicle and STZ 0 plus mAb did not reach statistical significance (STZ 0 plus vehicle vs. STZ 0 plus mAb n.s. = *p* > 0.999, *p* = 0.3152). 

Taken together, the present data indicated that the hippocampus of STZ-infused mice undergoes abnormal N-terminal tau cleavage along with other classical biochemical signs of disease (up-regulation of APP, BACE-1 and site-specific tau hyperphosphorylation) that can be successfully restored by non-invasive delivery of the cleavage-specific 12A12mAb. 

### 2.3. 12A12mAb Immunization Improves the STZ-Induced Neuroinflammation and Restores the PI3K/AKT/GSK3-β and AMPK Signaling Pathways

**Figure 5:**
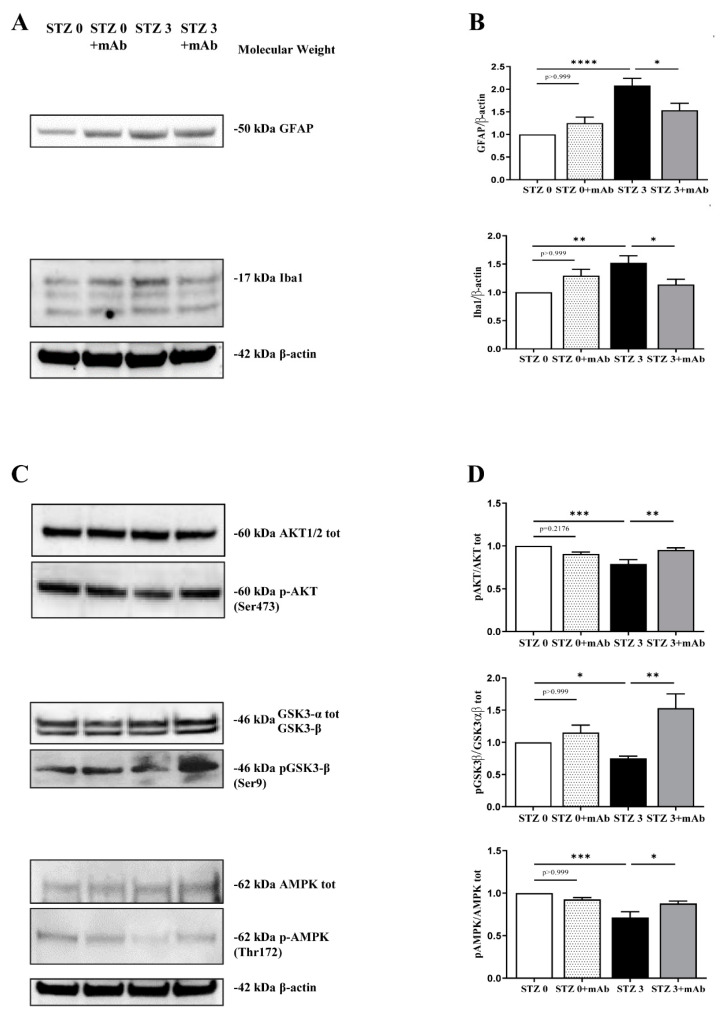
Effect of 12A12mAb immunization on cerebral neuroinflammation and dysregulation in several signal transduction pathways underlying the AD-like phenotype of ICV-STZ mice. (**A**–**C**) Hippocampal homogenates from animals of four experimental groups (STZ 0 plus vehicle; STZ 0 plus mAb; STZ 3 plus vehicle; STZ 3 plus mAb) were analyzed by Western blotting by developing with antibodies reported alongside the blots. (**B**–**D**) Semi-quantitative densitometry of the intensity signals of bands was carried out following normalization with β-actin level used as loading control. Arrows on the right side indicate the molecular weight (kDa) of bands calculated from migration of standard proteins. Values are from at least three independent experiments and statistically significant differences were calculated by one-way ANOVA followed by Bonferroni’s post-hoc test for multiple comparison among more than two groups. *p* < 0.05 was accepted as statistically significant (* *p* < 0.05; ** *p* < 0.01; *** *p* < 0.0005; **** *p* < 0.0001).

Neuroinflammation with astrogliosis and microglial activation is a well-established hallmark of sAD brain [44]. Thus, we determined the expression levels of GFAP, a marker of astrocytes, and Iba1, a marker of microglia, on hippocampal protein extracts from animals of four experimental groups (STZ 0 plus vehicle; STZ 0 plus mAb; STZ 3 plus vehicle; STZ 3 plus mAb) by SDS-PAGE Western blotting analysis. As shown in Figure 5A,B, significant differences in GFAP and Iba-1 immunoreactivity were found in STZ-infused mice (STZ 3 plus vehicle vs. STZ 0 plus vehicle, ** *p* < 0.01; **** *p* < 0.0001), in line with previous investigations in the ICV-STZ mouse model [12,23,29,34]. Of note, i.v. delivery of 12A12mAb markedly reduced the activation of both inflammatory markers in STZ-infused mice when compared to their not-immunized controls (STZ 3 plus vehicle vs. STZ 3 plus mAb, * *p* < 0.05). Likewise, the antibody treatment was ineffective in altering the proteins expression when STZ 0 plus vehicle mice were compared with antibody-treated counterparts (STZ 0 plus vehicle vs. STZ 0 plus mAb n.s. = *p* > 0.999).

The serine-threonine protein kinase B (AKT1/2) and GSK3-β are involved in the brain insulin/insulin-like growth-factor-1 (IGF-1) signaling whose dysregulation correlates with the severity of dementia symptoms in sAD [45]. In particular, since the binding of insulin to the insulin receptor (IR) leads to the activation of PI3K/AKT and inactivation of GSK3-β, any disturbance in this transduction pathway will result in insulin resistance. Additionally, GSK3-β is causally linked with the amyloidogenesis, the abnormal tau hyperphosphorylation at multiple sites and with inflammatory activation, representing thus an important hub between diabetes mellitus (DM) and AD which is also regarded as Type 3 diabetes (T3D) [46,47]. Thus, we also checked for the activation status of crucial downstream components of this pathway by evaluating the phosphorylation levels of AKT1/2(Ser473) and GSK3-β(Ser9) in hippocampal extracts of mice, expressed as ratio of *p*-AKT(Ser473)/AKT1/2 and pGSK3-β(Ser9)/GSK3-α/β immunoblot bands. The complete activation of upstream AKT1/2 requires its phosphorylation at Ser473 site while GSK3-β becomes inactive when it is phosphorylated at Ser9 residue by activated AKT1/2. As shown in Figure 5C,D, the relative intensities of *p*-AKT/AKT1/2 and pGSK3-β/GSK3-α/β signals were significantly decreased following ICV-STZ in treated mice as compared with the control group (STZ 3 plus vehicle vs. STZ 0 plus vehicle, ** *p* < 0.01; * *p* < 0.05), in line with previous findings [12,30,48]. No difference in the total AKT1/2 and GSK3-β levels was found in any group (*p* > 0.999). However, immunization with 12A12mAb elevated this ratio in STZ-infused mice when compared to non-immunized controls, indicating that antibody delivery effectively decreased the in vivo activity of GSK3-β via the stimulation of AKT1/2-dependent inhibitory phosphorylation (STZ 3 plus vehicle vs. STZ 3 plus mAb, ** *p* < 0.01; *** *p* < 0.0005).

Another important feature of ICV-STZ mice in parallel with brains from sAD patients is the down-regulation of AMP-activated protein kinase (AMPK) activity, evaluated as its phosphorylation state at Thr172 site located on its α enzyme subunit [49]. AMPK is a serine/threonine kinase whose activation attenuates the Aβ accumulation in AD, normalizes tau hyperphosphorylation and regulates insulin sensitization [50,51]. Therefore, we also measured the expression of *p*-AMPK and AMPK by quantifying the pAMPK(Thr172)/AMPK ratio in immunoblot bands. As shown in Figure 5C,D, the relative immunoreactivity level of pAMPK(Thr172)/AMPK signal was decreased in the hippocampus of STZ 3 plus vehicle mice when compared with their untreated counterpart (STZ 3 plus vehicle vs. STZ 0 plus vehicle *** *p* < 0.0005), in line with previous findings [49]. No difference in the total AMPK levels was found in any group (*p* > 0.999). Interestingly, when naïve groups were compared to their non-immunized controls, we found out that 12A12mAb immunization in STZ-infused mice significantly restored the activation of AMPK by up-regulating its Thr172 phosphorylation (STZ 3 plus vehicle vs. STZ 3 plus mAb, * *p* < 0.05). These findings are consistent with previous in vivo results showing that modulation of AMPK activity in the hippocampus of both STZ and APP/PS1 mice ameliorates the AD-like pathology and spatial memory dysfunction [31].

Taken together, all these behavioral and biochemical/molecular results indicated that 12A12mAb delivery was neuroprotective on sAD-phenotype of ICV-STZ mice by improving the cognitive function and several crucial features associated with neuropathology (activation of amyloidogenic pathway, tau hyperphosphorylation and neuroinflammation), likely via modulation of the AKT/GSK3-β and AMPK signaling pathways.

### 2.4. Overt Oxidative Stress and Mitochondrial Function Alterations Induced by ICV-Infusion of STZ Are Both Prevented by In Vivo 12A12mAb Delivery

Overt oxidative stress and owing to an imbalance between oxidants derivatives production and antioxidants defenses is another neurodegenerative AD-like hallmark observed in the STZ-infused animal model [14,15,16,52,53,54,55]. Although the exact mechanism of STZ-induced cytotoxicity is still not clear, significant decrease in respiratory chain complex activities and energy metabolism are detected when mice undergo injection of this compound [13,14,53,54], just recapitulating the causal involvement of mitochondrial deficits in onset/progression of AD pathophysiology. In addition to hippocampus, other brain regions are also strongly susceptible to diabetogenic insults [56,57] and the AMPK/SIRT/PGC-1α signaling pathway along with antioxidant capabilities have been recently reported to be down-regulated in cortical tissues of STZ-injected mice [58]. Thus, to further support our behavioral and biochemical findings, we specifically examined the metabolic changes in the oxidative stress induced by an enhanced cerebral generation of reactive oxygen species (ROS), which leads to mitochondrial dysfunction and cell redox unbalance. To this aim, into cortices from the four experimental groups, we measured the generation of ROS, whose overproduction causes membrane lipid peroxidation, and then analyzed the intracellular levels of GSH, NADH and NADPH which play critical roles in defining the activity of energy-producing pathways and in maintaining antioxidant defenses. 

**Figure 6:**
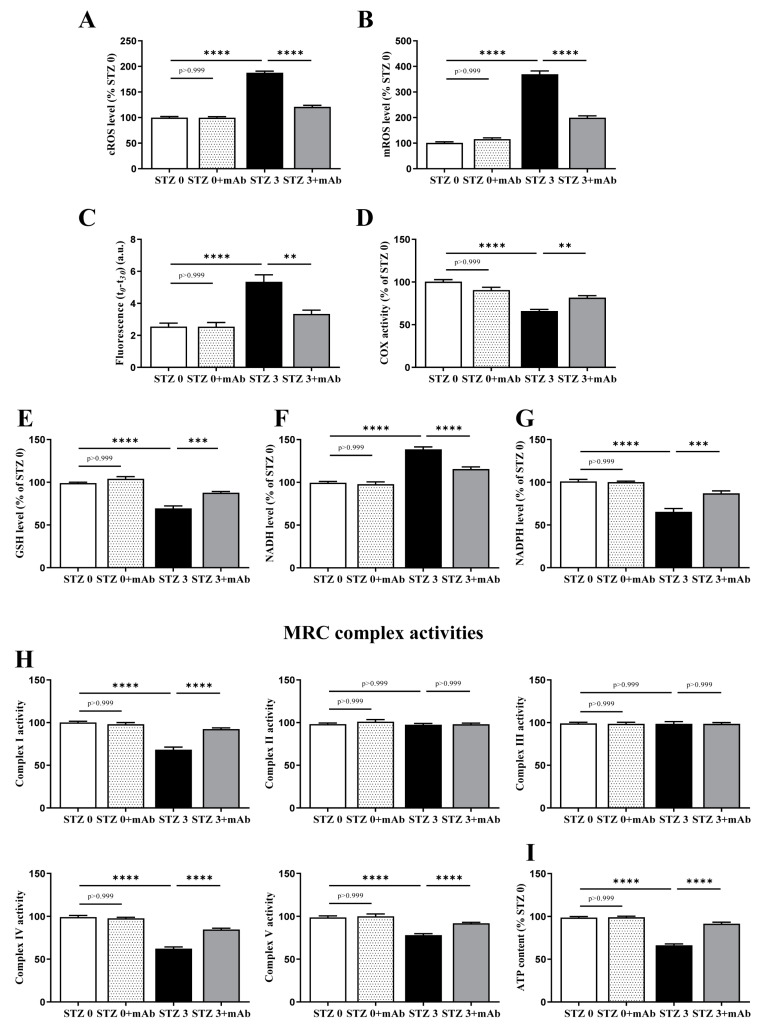
Overt oxidative stress and mitochondrial deficits occurring in brains from ICV-STZ mice are recovered by 12A12mAb immunization. (**A**–**I**) Cortical homogenates from animals of four experimental groups (STZ 0 plus vehicle; STZ 0 plus mAb; STZ 3 plus vehicle; STZ 3 plus mAb) were assessed for oxidative stress and oxidative phosphorylation. In (**A**,**B**): ROS level. Cytosolic and mitochondrial O2^−•^ productions were detected according to the adrenochrome method (**A**) and by using the MitoSOX dye (**B**), respectively. The O2^−•^ level value was expressed as percentage of STZ 0 plus vehicle, to which value 100 was given. In C–D: Lipid peroxidation and COX activity. Index of lipid peroxidation (**C**) are expressed as fluorescence values at the beginning of the reaction (t0) minus the value measured after 30 min (t30), accordingly to [66]. COX activity (**D**) was determined by measuring polarographically O2 ^− •^ consumption in the oxygen electrode chamber [74]. In (**E**–**G**): GSH, NADH and NADPH level determinations were calculated as in [68]. In (**H**): MRC complex activities: the activities of Complex I (NADH:ubiquinone oxidoreductase), Complex II (succinate:ubiquinone oxidoreductase), Complex III (cytochrome c reductase), Complex IV (cytochrome c oxidase) and Complex V (ATP synthase) [39]. In (**I**): Cellular ATP content [39]. Values are from at least three independent experiments and statistically significant differences were calculated by one-way ANOVA followed by Bonferroni’s post-hoc test for multiple comparison among more than two groups. *p* < 0.05 was accepted as statistically significant (** *p* < 0.01; *** *p* < 0.0005; **** *p* < 0.0001).

As shown in Figure 6A, by using epinephrine as cytosolic ROS detecting method, STZ-infused mice showed a cROS level doubled in comparison with the control group (STZ 3 plus vehicle vs. STZ 0 plus vehicle, **** *p* < 0.0001), in line with previous investigations [59,60,61]. Interestingly, the cellular level of ROS was markedly diminished in ICV-STZ 3 mice following the immunization of the cleavage-specific anti-tau 12A12mAb (STZ 3 plus vehicle vs. STZ 3 plus mAb, **** *p* < 0.0001). Likewise (Figure 6), by measuring the MitoSOX oxidation-dependent O2^−•^ level, we observed that mitochondrial ROS production (mROS) was about four-fold higher in STZ 3 homogenate when compared with non-STZ-infused mice (STZ 3 plus vehicle vs. STZ 0 plus vehicle, **** *p* < 0.0001) and halved in STZ 3 mice treated with 12A12mAb (STZ 3 plus vehicle vs. STZ 0 plus vehicle, **** *p* < 0.0001), although it still remained significantly higher than in STZ 0 mice plus mAb. No significant MitoSOX oxidation occurred when STZ 3 homogenate was incubated in the absence of SUCC + ADP (not shown). Additionally, no changes were detected in both cROS and mROS levels in STZ 0 plus vehicle group (*p* > 0.999, Figure 6A,B), indicating that the in vivo antibody delivery was not toxic. 

Excessive oxidative stress is tightly bound to mitochondrial dysfunction, as these organelles are both generators of and targets for ROS [62,63]. Thus, since severe oxidative stress resulting from O2^−•^ overproduction can damage the mitochondrial membranes via lipid peroxidation, we also explored this possibility in STZ cortex homogenate by using cis-Parinaric acid (PnAc), which readily incorporates into membranes and loses fluorescence as it becomes oxidized [64]. As shown in Figure 6C, the index of lipid peroxidation—calculated as the fluorescence value at the beginning of the reaction (t0) minus the value measured after 30 min (t30)—was about 2.0-fold higher in STZ 3 plus vehicle group in comparison with control one (STZ 3 plus vehicle vs. STZ 0 plus vehicle, **** *p* < 0.0001) but significantly reduced about approximately 40% following 12A12mAb injection (STZ 3 plus vehicle vs. STZ 3 plus mAb, ** *p* < 0.01). Again, no change was found when antibody was delivered to STZ 0 mice (*p* > 0.999). 

After that, we measured endogenous COX activity by following (ASC + TMPD)-dependent oxygen consumption because the activity of integral membrane proteins is known to be highly dependent on the lipid environment and peroxidative damage of mitochondrial membranes can negatively affect the activity of respiratory Complex IV (COX) [65]. Results in Figure 6D clearly show a decrease in Complex IV activity in STZ-injected mice (STZ 3 plus vehicle vs. STZ 0 plus vehicle, **** *p* < 0.0001), supporting the hypothesis that the ROS-mediated damage of the membrane microenvironment could be responsible for the observed inhibition of Complex IV. Following 12A12mAb immunization, the COX activity was partly rescued (STZ 3 plus vehicle vs. STZ 3 plus mAb, ** *p* < 0.01), suggesting that pathogenic tau could exert in vivo on neuronal mitochondria a mechanism similar to that which occurs in vitro following exposure of toxic Aβ1-42 peptide(s) [66].

Since the mitochondrial dysfunction and the generation of ROS are intricately related to changes in the antioxidant scavenging system [67], the levels of GSH/GSSG and the redox states of the NAD and NADP^+^ pyridine nucleotide pools were next investigated. The cell redox balance depends mainly on the ratios of the oxidized and reduced partners of the redox pairs NADH/NAD^+^, NADPH/NADP^+^ and GSH/GSSG, which play critical roles in defining the activity of energy-producing pathways and in maintaining the antioxidant defenses. Under normal conditions, the NADH/NAD^+^ pair is predominantly in the oxidized state to accept electrons produced during glycolysis; whereas, the redox pairs, NADPH/NADP^+^ and GSH/GSSG, are biased towards the reduced state to provide electrons for reductive biosynthesis and anti-oxidative processes, respectively. Furthermore, considering that the glycolysis and the Krebs cycle and the respiratory chain are functionally closely coupled, any detected defects of mitochondrial respiration (Figure 6B) may lead to NADH level increase being its oxidation impaired in mitochondrial respiration [68]. As shown in Figure 6E,F and in agreement with previous findings [55], a drop of GSH levels (about 30%) was clearly found in the brain of ICV-STZ-treated mice (STZ 3 plus vehicle vs. STZ 0 plus vehicle, **** *p* < 0.0001) whereas the cytosolic redox state of the NADH/NAD^+^ pair was biased towards the reduced state (STZ 3 plus vehicle vs. STZ 0 plus vehicle, **** *p* < 0.0001). Additionally, the pro-oxidant shift of the NADPH/NADP^+^ pair—which provides the principal cellular reducing equivalents required by many antioxidant defense systems [69,70]—was also observed following ICV-STZ (STZ 3 plus vehicle vs. STZ 0 plus vehicle, **** *p* < 0.0001), suggesting that the reduced availability of NADPH could be responsible, at least in part, for the low intracellular GSH level leading to the overproduction of free radical levels. Interestingly, the GSH, NADPH and NADH levels were largely normalized following administration of 12A12mAb (STZ 3 plus vehicle vs. STZ 3 plus mAb, *** *p* < 0.0005; **** *p* < 0.001). Again, antibody treatment was ineffective per se in altering the GSH, NADPH and NADH levels when STZ 0 plus vehicle mice were compared with their antibody-treated counterpart (STZ 0 plus vehicle vs. STZ 0 plus mAb, *p* > 0.999).

To explore whether the increase of ROS and NADH levels could be due to the inhibition of one or more mitochondrial chain complexes [71,72,73], we examined the activity of each of the five complexes, including the ATP synthase (Figure 6H) and the cellular ATP content (Figure 6I). A decrease in activities of Complex I, IV and V (normalized to the total homogenate protein content) and in the ATP levels was clearly detected (Figure 6H,I, respectively) in the brain of ICV-STZ-treated mice (STZ 3 plus vehicle vs. STZ 0 plus vehicle, **** *p* < 0.0001) and these changes were reversible in a 12A12mAb-dependent manner (STZ 3 plus vehicle vs. STZ 3 plus mAb, **** *p* < 0.0001). No differences in the activities of Complexes II and III were observed among the four experimental groups (*p* > 0.999). Again, antibody treatment was ineffective per se in altering the Complex I, IV and V levels and the overall amount of *p* when STZ 0 plus vehicle mice were compared with their antibody-treated counterpart (STZ 0 plus vehicle vs. STZ 0 plus mAb, *p* > 0.999). 

Taken together, these findings demonstrated that in the brains of STZ-infused mice, the 12A12mAb delivery exerted a protective action against and OXPHOS impairment and oxidative stress by mitigating the increase in the cytosolic and mitochondrial ROS, the lipid peroxidation of the mitochondrial membrane responsible for the impairment of COX activity, as well as the decrease in the levels of GSH and NADPH and the increase in NADH level.

### 2.5. 12A12mAb Administration Normalizes the Imbalanced Expression of Key Synaptic Proteins in the Hippocampus of STZ-Lesioned Mice

In addition to mitochondrial impairment, synaptic deterioration is another early event involved in sAD pathogenesis with alterations in pre- and postsynaptic proteins being recognized as a core feature correlating with deficits in synaptic plasticity and cognitive decline [75,76]. In line, STZ-induced mice exhibit significant damage of hippocampal neurons and synapses [36,77,78,79,80].

**Figure 7:**
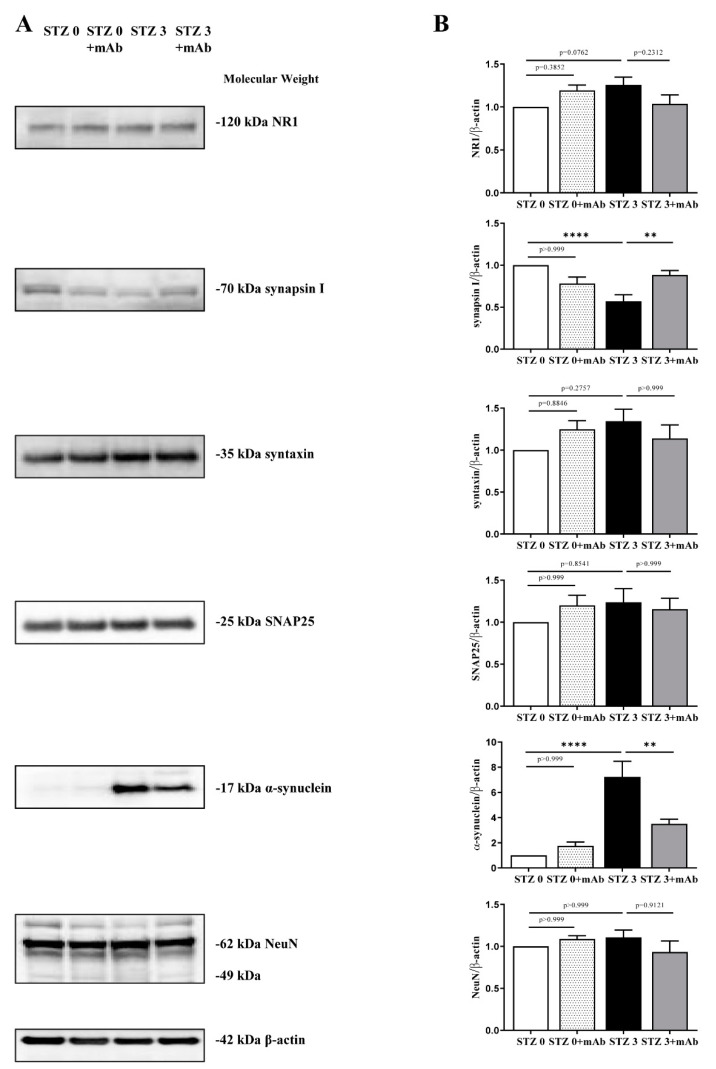
STZ-induced imbalanced expression of key synaptic proteins in hippocampus is normalized following 12A12mAb administration. (**A**) Hippocampal homogenates from animals of four experimental groups (STZ 0 plus vehicle; STZ 0 plus mAb; STZ 3 plus vehicle; STZ 3 plus mAb) were analyzed by Western blotting by developing with antibodies against several synaptic, including synapsin I, SNAP25 α-synuclein, syntaxin, N-Methyl-d-aspartate (NMDA) receptor subunit NR1, NeuN. (**B**) Semi-quantitative densitometry of the intensity signals of bands was carried out following normalization with β-actin level used as loading control. Arrows on the right side indicate the molecular weight (kDa) of bands calculated from migration of standard proteins. Values are from at least three independent experiments and statistically significant differences were calculated by one-way ANOVA followed by Bonferroni’s post-hoc test for multiple comparison among more than two groups. *p* < 0.05 was accepted as statistically significant; ** *p* < 0.01; **** *p* < 0.0001).

Thus, by Western blotting SDS-PAGE analyses carried out on hippocampal protein extracts from four experimental groups (STZ 0 plus vehicle; STZ 0 plus mAb; STZ 3 plus vehicle; STZ 3 plus mAb), we also measured the steady-state levels of several relevant synaptic markers, including synapsin I, SNAP25, α-synuclein, syntaxin, N-Methyl-d-aspartate (NMDA) receptor subunit NR1. As shown in Figure 7A,B, we observed that ICV-STZ treatment induced a reduction of synapsin I in concomitance of strong increase of α-synuclein immunoreactivities (STZ 3 plus vehicle vs. STZ 0 plus vehicle, **** *p* < 0.0001), both of them being largely prevented by 12A12mAb immunization (STZ 3 plus vehicle vs. STZ 3 plus mAb, ** *p* < 0.01). No statistically significant differences in the intensity bands of the other analyzed synaptic proteins—SNAP25, syntaxin, N-Methyl-d-aspartate (NMDA) receptor subunit NR1—were detected in the hippocampi from all four experimental groups (*p* > 0.999, *p* = 0.8846, *p* = 0.3852). Likewise, no change was detected in the expression of neuronal marker NeuN, indicating that these synaptic alterations occurred in STZ-injected mice in the absence of overt neuronal loss.

**Figure 8:**
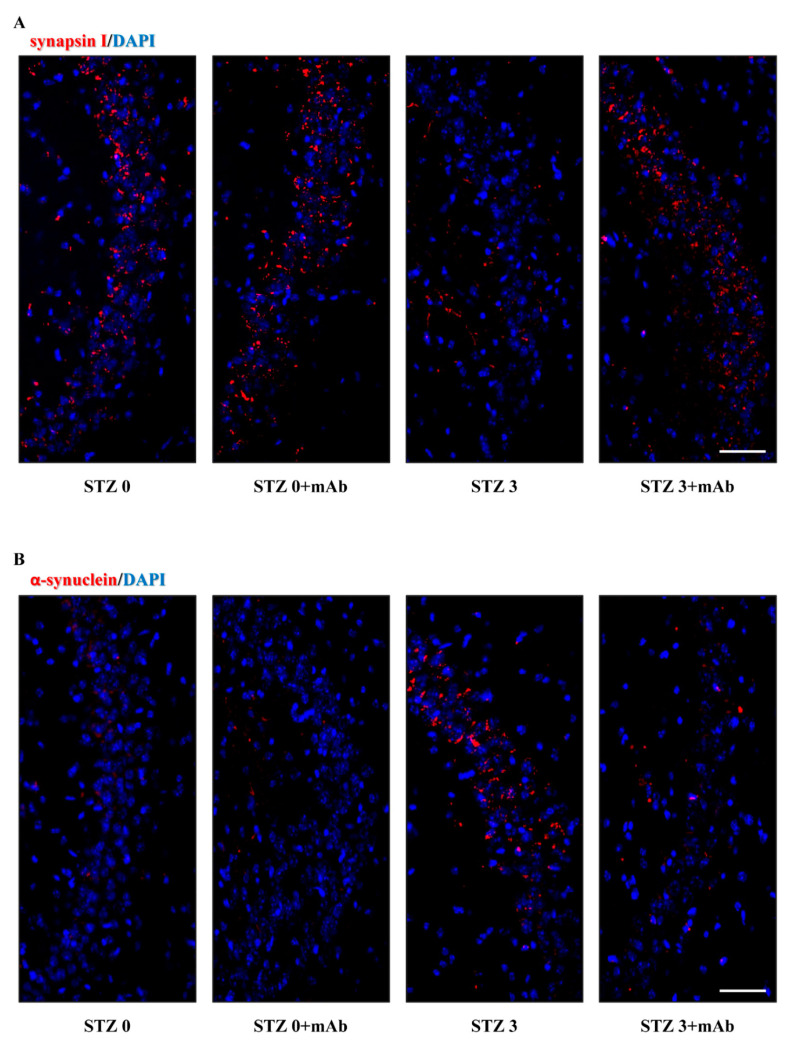
Effect of 12A12mAb immunization on synaptic changes of synapsin I and α-synuclein in the hippocampus of ICV-STZ mice. (**A**,**B**) Representative images (n = 3) of immunofluorescence analysis (20×) showing the punctate staining of the synapsin I (**A**) and α-synuclein (**B**) (red channel) in hippocampal CA2/CA3 regions from animals of four experimental groups (STZ 0 plus vehicle; STZ 0 plus mAb; STZ 3 plus vehicle; STZ 3 plus mAb). Nuclei were counterstained with DAPI (blue channel). Scale bar = 20 µm.

To further confirm the influence of tau immunization on synaptic protein expression, on sagittal sections of animals’ hippocampi we performed immunofluorescence evaluations of the two markers, synapsin I and α-synuclein that we found out to be altered following STZ injection. Consistent with biochemical results, confocal microscopy analysis (Figure 8A,B) displayed that the basal levels of these two proteins were differently modulated following STZ injection in mice and that both of them respond to delivery of 12A12mAb. In particular, in the CA2 and CA3 subregions known to be largely affected following STZ infusion [80,81], the dot-like labeling of synapsin I (Figure 8A) was down-regulated in STZ-treated group (STZ 3 plus vehicle) when compared with both controls (STZ 0 plus vehicle, STZ 0 plus mAb) and its staining was significantly recovered following 12A12mAb administration (STZ 3 plus mAb). Conversely, in comparison with both controls (STZ 0 plus vehicle, STZ 0 plus mAb), α-synuclein expression appeared to be markedly up-regulated in mice following STZ injection (STZ 3 plus vehicle) and then, normalized by 12A12mAb delivery (STZ 3 plus mAb). 

Taken together, these findings indicated that: (i) ICV-STZ caused deregulation of selected synaptic proteins which might disturb synaptic plasticity and, then, underlie the cognitive impairment in treated mice; (ii) the neuroprotection offered by 12A12mAb delivery on STZ-induced memory/learning deficits was associated with improved hippocampal synapses.

### 2.6. STZ-Induced Histopathologic Alterations Are Improved in the Hippocampus of Injected Mice following 12A12mAb Immunization

**Figure 9:**
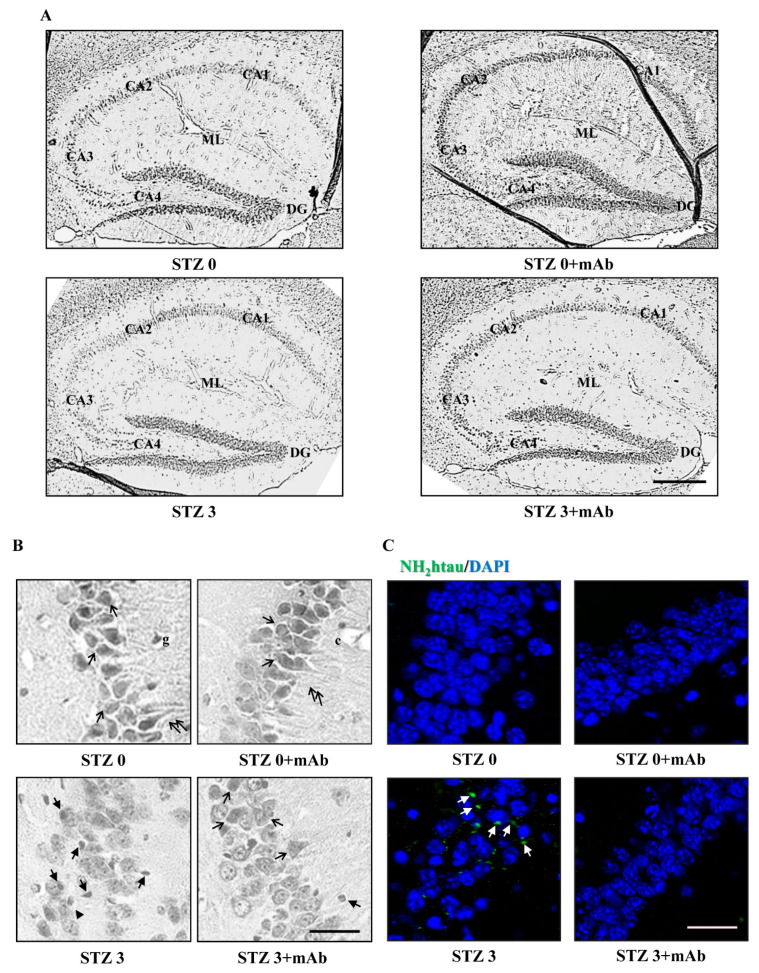
Histopathological alterations in the hippocampus of ICV-STZ mice are mitigated following 12A12mAb-mediated neutralization of the NH_2_htau. (**A**) Representative images of hippocampal slices showing hematoxylin and eosin staining under examination with a light electric microscope at 4×. Scale bar = 50 μm. (**B**) 40× magnification of CA3 subfield. Notice that STZ alters the laminar organization and significantly increases the number of degenerating neurons with apoptotic disintegration of the nucleus (black arrows with heads). Following 12A12mAb treatment, neuroprotection is evident as shown by the presence of well-preserved hippocampal cyto-architecture characterized by viable neurons with healthy, not-damaged nuclei (arrows). Scale bar = 10 μm. (**C**) Immunofluorescence analysis (n = 4) at 40× magnification showing the distribution/expression of the NH_2_htau peptide (green channel) in hippocampal CA3 regions from animals of four experimental groups (STZ 0 plus vehicle; STZ 0 plus mAb; STZ 3 plus vehicle; STZ 3 plus mAb). Nuclei were counterstained with DAPI (blue channel). Notice that, unlike not-immunized mice, the laminar organization/integrity is well preserved in STZ-treated hippocampi following 12A12mAb treatment in correlation with a significant diminution in signal of the NH_2_htau (white arrows). Scale bar = 10 µm.

To assess the morphological changes in hippocampus occurring under our experimental conditions, we examined hematoxylin and eosin (H&E)-stained slides of sagittal sections from animals of four experimental groups (STZ 0 plus vehicle; STZ 0 plus mAb; STZ 3 plus vehicle; STZ 3 plus mAb). As shown in Figure 9A, the typical C-shaped pattern of hippocampus appeared to be well-preserved in sections from both control groups (STZ 0 plus vehicle, STZ 0 plus mAb) showing areas with compact cytoarchitecture in the four regions (CA1, CA2, CA3 and CA4), the dentate gyrus (DG) and the underneath molecular layer (ML). On the contrary, in sections from STZ-lesioned animals (STZ 3 plus vehicle), pyramidal layer of hippocampal structure appeared to be disordered with many areas of cell loss, indicating that STZ was able to cause damage to the brain of mice. Relevantly and in agreement with behavioral results showing an impaired learning and memory function (Figure 2 and Figure 3), 12A12mAb induced an evident preservation of large pyramidal neurons of treated mice, especially in CA3 subfield shown to be markedly damaged following STZ infusion [80,81].

In CA3 region magnification (Figure 9B), large pyramidal cells (black arrows) and molecular layer with the intact processes of neurons (black double arrows), glial cells (g) and capillaries (c) were clearly evident in both control groups (STZ 0 plus vehicle, STZ 0 plus mAb). Examination of STZ 3 plus vehicle group revealed complete disorganization and dispersion of pyramidal cells with many degenerating neurons appearing scattered and deformed with vacuolated cytoplasm and fragmented, hyperchromatic, dark pyknotic or karyolitic nucleus (black arrows with heads) whereas some having a halo around them (black arrowheads). A marked improvement in neuropathology was discernable in STZ 3 plus mAb group, indicating that 12A12mAb administration significantly reduced signs of overt neurodegeneration in vivo. 

Furthermore, to confirm the involvement of the NH_2_htau in triggering neuronal injury, hippocampal sections from the four experimental groups were analyzed by immunofluorescence staining with caspase-cleaved protein (CCP)-NH_2_tau antiserum (D_25_-(QGGYTMHQDQ) epitope), as we previously reported [39,82,83]. A strong dot-like positivity was detected in degenerating neurons endowed with pyknotic nuclei (white arrows) from the most compromised CA3 area of STZ 3plus vehicle group (Figure 9C). On the contrary, no labeling was clearly detectable either in both control groups (STZ 0 plus vehicle, STZ 0 plus mAb) or in 12A12mAb-treated STZ-lesioned mice (STZ 3 plus mAb group), further corroborating the H&E result.

Collectively, these results showed that some histopathological alterations typically detected in hippocampus of ICV-STZ mice [84,85] are significantly ameliorated in vivo by 12A12mAb-mediated neutralization of the toxic, endogenously generated NH_2_htau. 

## 3. Discussion

A large number of drug candidates, showing effective anti-AD actions in preclinical studies, ended up failing when tested in clinical trials, in part due to the inappropriate selection of the animal model recapitulating the sporadic form of the disease (sAD), which represents about 95% of all affected cases. Familial AD (fAD) and sAD share many neuropathological features, including Aβ accumulation and toxicity, tau hyperphosphorylation, oxidative stress, neuroinflammation, cholinergic neuronal degeneration, activation of apoptotic pathways, insulin desensitization/resistance state, synapse alteration and autophagy dysfunction. However, other differentiating mechanisms—such as early vs. late onset of symptoms, preferential parietal atrophy and white matter abnormalities vs. more pronounced hippocampal volume loss and high incidence of diabetes and cardio-circulatory disorder, defects in clearance vs. overproduction of Aβ and others—may explain the reasons why potential anti-AD compounds seem to work in animal models but not in humans. 

Thus, it is needed to test the efficacy of potential AD therapies in a wide range of different (transgenic and non-transgenic) animal models that: (i) take into account the multifactorial and heterogeneous nature of the disorder, involving several etiopathogenic mechanisms; (ii) recapitulate the most common sporadic form (sAD) [86]. In this context, the intracerebroventricular administration of streptozotocin (ICV-STZ) to rodents is considered an appropriate model of sAD for the alternative and complementary investigation of therapeutic compounds [30,32,40]. Indeed, ICV-STZ mice develop neuroinflammation, amyloidogenesis, tau hyperphosphorylation, brain insulin resistance and cognitive impairments, just as reported to occur in human brains of affected subjects [12,33,34,41,46].

The main finding of the present study is that ICV-STZ mice develop AD-relevant N-terminal cleavage of tau, with the generation of toxic peptide(s), such as the 20–22 kDa fragment [38], in tight association with their cognitive impairment (assessed as NOR and OPR performance tasks) and with other key neurochemical (amyloidogenesis, tau hyperphosphorylation, gliosis, synaptic alterations assessed by Western blotting and immunofluorescence) and histological (cell loss/injury evaluated by hematoxylin and eosin staining) hallmarks. Consistent with the pathogenic role of the NH_2_htau peptide in this non-transgenic animal model of sAD, these experiments confirm and further advance our previous preclinical findings from two different lines of fAD transgenic mice, such as 6-month-old Tg2576 and 3xTg [1,39]. More importantly, the present study further validates the neuroprotective effects of targeting the neurotoxic NH_2_htau in vivo by non-invasive delivery of the cleavage-specific anti-tau 12A12mAb, pointing out its safe use for the medical treatment of AD. Interestingly, Food and Drug Administration (FDA)-approved treatments for AD, such as donepezil and memantine, are reported to improve STZ-induced AD-like phenotype and associated neuropathology corroborating the predictive validity of their efficacy in humans [87,88,89]. Our results also disclosed a time-dependent beneficial action of 12A12mAb delivery on STZ-induced cognitive deficits in recognition and spatial memory. Indeed, in STZ-lesioned mice undergoing immunization, both the level of exploration and the percentage of discrimination in NORT and OPRT performances are higher when evaluated after the long-term compared to the short-term regimen. These findings are consistent with a gradual increase in the intracellular steady-state expression level of the neurotoxic NH_2_htau we detected over time by Western blotting analysis on hippocampi from mice injected with STZ (3 mg/Kg) for 7, 14, 21 days (Figure S1). Besides, our biochemical results point out that the detrimental effects of toxic non-fibrillary tau species are reversible in vivo within a certain frame of time [90], when a biologically efficacy, therapeutic concentration of neutralizing antibody is reached. 

Inhibition of insulin receptor (IR) signaling with deregulation of phosphatidylinositol 3′ kinase–AKT–glycogen synthase kinase3 (PI3K–AKT–GSK3-β) pathway along with the development of Aβ and tau pathology have been reported in post-mortem brains of sAD patients [5,91,92,93] and in ICV-lesioned mice with sub-diabetogenic (up to 3 mg/Kg) doses of STZ [94,95,96]. Increased Aβ1-42 and hyperphosphorylated tau immunoreactivity, typically featuring the AD neuropathology in affected subjects, have also been detected in the hippocampus of STZ-infused rodents [12,18,94,97,98,99]. Additionally, the proline-directed kinase GSK3 is known to phosphorylate tau at canonical sites in the sAD brain [100] and to mediate amyloid β neurotoxicity [47]. GSK3 facilitates the production of amyloid β in brains of APP transgenic mice [101] and its inhibition reduces the amyloid β production [102]. Pharmacological modulation of (PI3K–AKT–GSK3-β) pathway improves the hippocampus-dependent memory impairment in STZ-lesioned rodents and in transgenic AD animal models undergoing tau pathology [46,103]. Our in vivo experiments on the neuroprotective action of 12A12mAb, including the down-regulation of the amyloidogenic pathway (i.e., APP and BACE-1 expression levels) and of the ptau/total tau ratio and the improvement in cognitive performance, involving the modulation of Ser9 phosphorylation of GSK3-β in brains of ICV-STZ mice, are consistent with these findings. 

We also found that STZ-induced astrogliosis and microgliosis, as evidenced by a reduction in GFAP and Iba-1 expression levels, are partly rescued by 12A12mAb immunization, in line with the findings that GSK3-β inactivation decreases the microglial migration and inflammatory response [104]. Finally, the evidence that 12A12mAb immunization rescues the cognitive impairments in ICV-STZ mice, in part by up-regulating the level of *p*-AMPK (active form), is also consistent with previous studies reporting a significant improvement of the hippocampal AD-like pathology and spatial memory deficits in this animal model of sAD following stimulation of AMPK with its specific activator AICAR [31]. Taken together, these findings are in good agreement with the evidence that another “gain-of-function” form of truncated tau involves a dysregulation of insulin signaling likely via the AKT/GSK3-β pathways. To this regard, it has been reported that overexpression of another pathogenic tau fragment, named C-terminal Tau35, disrupts the insulin responsiveness in Chinese hamster ovary (CHO) cell line by repressing both AKT activation and its inhibitory phosphorylation of GSK3-β [105]. The current study expands these in vitro results by reporting for the first time that the cleavage of tau at its N-terminal projection domain releases in ICV-STZ mice the AD-relevant toxic NH_2_htau [38], which can contribute to aggravate the cognitive deficits by inhibiting the AKT/GSK3-β signaling. 

In line with previous investigations [14,15,34,106,107,108], we also detected into brains of ICV-STZ mice a prominent increase in lipid peroxidation—which is generally used as an indicator of free radical overproduction—along with concomitant decline in antioxidant scavenger system(s) and mitochondrial impairment. Importantly, the 12A12mAb i.v. delivery exerted a protective action by mitigating the increase in both cytosolic and mitochondrial ROS, the lipid peroxidation of the mitochondrial membrane which is responsible for the impairment of COX activity, the decrease in the level of GSH and NADPH and the increase in NADH level, as well as the oxidative phosphorylation and the ATP content. Consistent with the pivotal role of ATP-producing mitochondria in energy-demanding synaptic terminals, the expression of two key synaptic proteins—such as synapsin I and α-synuclein known to be imbalanced in hippocampus of STZ-injected mice in association with poor behavioral performance [78,109]—is also normalized by in vivo neutralization of the NH_2_htau. Additionally, our morphological results combining hematoxylin and eosin (H&E) staining and immunofluorescence analysis also confirmed that a significant increase in the number of darkly stained, apoptotic neurons was evident in different hippocampal regions of STZ-lesioned mice, as reported by previous histopathological investigations [84,85], and that neutralization of the endogenously generated toxic NH_2_htau following 12A12mAb immunization provided a marked in vivo protection from cell degeneration. These findings indicate that the neuroprotective effect of tau immunization involves, in part, the modulation of oxidative stress and mitochondrial energetic deficits, which are known to crucially contribute to the age-related synaptic decline of sAD in humans [110].

From a translational point of view, it is worth stressing that pre-clinical animal models of AD are incomplete [111], as they do not faithfully recapitulate the entire and dynamic spectrum of cognitive and non-cognitive manifestations associated with disease phenotype as observed in human conditions. Nevertheless, the finding that in vivo delivery of 12A12mAb offers a significant beneficial action on several key neurochemical, metabolic, anatomo-pathological and cognitive AD-like features, which are commonly shared by different but complementary—both genetic (i.e., Tg2576, 3xTg) and experimentally-induced (i.e., ICV-STZ)—mouse strains strongly support its future translation for the medical treatment of suffering patients. 

**Figure 10:**
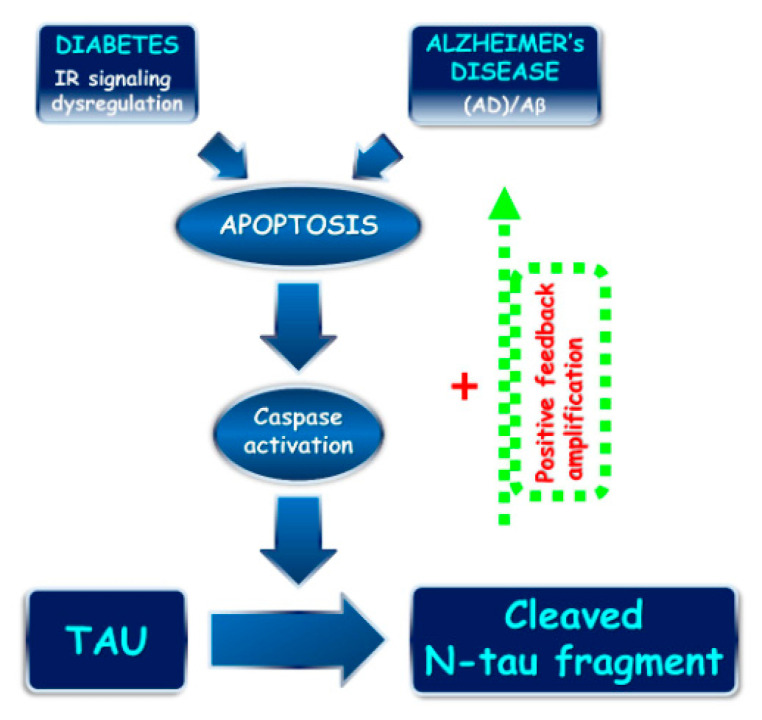
N-terminal tau truncation: a common mechanism linking sporadic AD pathogenesis and diabetes. Proposed role of N-terminal tau cleavage correlating sAD and diabetes. An imbalance in IR signaling (diabetes) and APP/Aβ dysmetabolisms (AD) trigger the apoptotic pathway, including caspase(s) activation [42,112] and consequent N-terminal tau truncation with generation of the 20–22 kDa NH_2_htau peptide. The NH_2_htau promotes the APP/Aβ misprocessing [115,116] and perpetuates/amplifies the neurodegenerative process by means of feedback forward mechanism.

At the mechanistic level, we can envisage that the STZ treatment induces in the brain an early activation of apoptotic pathway(s) eliciting the caspase(s)-dependent cleavage of tau with release of one and/or more toxic fragment(s), including the N-terminal tau peptide (NH_2_htau) which, in turn, might directly and/or indirectly affect neuronal functions and signalings (Figure 10). We suggest that the deleterious actions exerted by modest but statistically significant increase of the intracellular level of NH_2_htau into hippocampus of STZ-lesioned mice spread along a feedforward mechanism, which is successfully antagonized by specific neutralization following treatment with 12A12mAb resulting, thus, in a robust neuroprotection. In support of this hypothesis, it has been reported that hyperglycemia induces pathological tau truncation into a smaller product(s) around 25 kDa both in vitro, after exposure of primary cortical neurons to high glucose, and in vivo, in the brains of *ob*/*ob* type 2 diabetic mice [42,112]. Additionally, hippocampal neurons of both STZ-infused [29,31] and diabetic mice [113] have been shown to express apoptotic markers, such as TUNEL staining and cleaved caspase-3 activation. In vitro, in rat retina cell line [114] and, in vivo, after intranasal delivery in STZ-lesioned mice [29], treatment with insulin provides strong neuroprotection via modulation of the AKT/GSK3-β pathway and inactivation of caspase-3. In this framework, 12A12mAb, might be a therapeutic candidate both for subjects suffering from sAD and for patients with diabetes whose comorbidity contributes to the development of sAD.

## 4. Conclusions

Taken together, these in vivo findings indicate for the first time that tau-directed immunotherapy offers strong protection against cognitive dysfunction, oxidative stress, neurochemical and histological alterations occurring in STZ-induced mouse model of AD, which highlights the potential of non-invasive administration of 12A12mAb in the treatment of both familial and sporadic form of this age-related progressive neurodegenerative disorder.

## 5. Materials and Methods

### 5.1. Animals

All animal procedures and experiments were carried out in compliance with ARRIVE guidelines and with the European Guidelines for the use of animals in research (2010/63/EU) and in accordance with the Italian Ministry of Health (D.L. 26/2014). All procedures were approved by the internal animal welfare office and by the Department of Public Health and Veterinary. C57BL/6J male mice (bred and reared in the Santa Lucia Foundation (FSL) facility in Rome, Italy) were housed in a standard 12-h light/dark cycle at constant temperature and humidity, in a virus/antigen-free facility and subjected to experiments at 6 months of age. Food and water were always provided ad libitum. Experimental approval was obtained from the Italian Ministry of Health (approval No.691/2015-PR).

### 5.2. Chemical Compounds and Antibodies

STZ (2-deoxy-2-(3-(methyl-3-nitrosoureido)-D-glucopyranose) was purchased from Sigma-Aldrich, St. Louis, MO, USA. 

The following antibodies were used: tau antibody (BT2) mouse MN1010 ThermoFisher Scientific (Waltham, MA, USA); caspase-cleaved protein (CCP) NH_2_-tau antibody rabbit (D_25_-(QGGYTMHQDQ) epitope, phosphorylation-independent state) [82]; anti-Alzheimer precursor protein 22C11 (66–81aa of N-terminus) mouse APP-MAB348 Chemicon (Temecula, CA, USA); BACE-1 (61-3E7) mouse sc-33711 Santa Cruz Biotechnology (Dallas, TX, USA); anti-poly-tau (243-441aa) rabbit A0024 Dako (Santa Clara, CA, USA); anti-phospho-tau (pSer199/202) rabbit T6819 Sigma-Aldrich (St. Louis, MO, USA); anti-AKT antibody rabbit 9272 Cell Signaling Technology (Danvers, MA, USA); anti-phospho-AKT (Ser473) antibody rabbit 9271 Cell Signaling Technology (Danvers, MA, USA); anti-GSK3α/β (0011-A) mouse sc- 7291 Santa Cruz Biotechnology (Dallas, TX, USA); anti-phospho-GSK3-β (Ser9) antibody rabbit 9336 Cell Signaling Technology (Danvers, MA, USA); anti-phospho-AMPKα (Thr172) antibody rabbit 2531 Cell Signaling Technology (Danvers, MA, USA); anti-AMPK antibody rabbit 2532 Cell Signaling Technology(Danvers, MA, USA); GFAP antibody (2E1) mouse sc-33673 Santa Cruz Biotechnology (Dallas, TX, USA); Iba1 antibody (1022-5) mouse sc-32725 Santa Cruz Biotechnology (Dallas, TX, USA);NMDAζ1 antibody (C-20) goat sc-1467 Santa Cruz Biotechnology ((Dallas, TX, USA); anti-synapsin I antibody rabbit AB1543P Millipore (Burlington, MA, USA); anti-syntaxin 1 mouse S1172 Sigma-Aldrich (St. Louis, MO, USA); anti-SNAP25 antibody (clone SMI 81) mouse 836301 BioLegend (San Diego, CA, USA); anti-α-synuclein antibody (clone 42) mouse 610786 BD Transduction Laboratories (Franklin Lakes, USA, NJ); anti-α-synuclein antibody rabbit (D37A6) XP Cell Signaling Biotechnology (Danvers, MA, USA); Anti-NeuN antibody (clone A60) mouse MAB377 Millipore (Burlington, MA, USA); anti-β-actin antibody mouse S3062 Sigma-Aldrich (St. Louis, MO, USA); anti-mouse IgG (whole molecule)-Peroxidase antibody A4416 Sigma-Aldrich (St. Louis, MO, USA); anti-rabbit IgG (whole molecule)-Peroxidase antibody A9169 Sigma-Aldrich(St. Louis, MO, USA).

### 5.3. Neurosurgery and ICV-STZ Delivery

Mice were ICV bilaterally micro-infused in lateral ventricles with either STZ (3 mg/Kg in 3 μL/side) or citrate buffer (0.5 M, pH 4.5). The sub-diabetogenic dosage, administration time and route were performed according to previously published studies referring STZ-treated mouse as well-established sAD animal model [12,117]. Mice were anesthetized with 2.5% avertin (2,2,2 tribromoethanol; Sigma-Aldrich) by an intraperitoneal (i.p.) administration and next secured on a stereotaxic frame (Kopf apparatus). Stereotaxic coordinates from the Bregma were as follows: ±1.0 mm L, −0.5 mm AP and −2.5 mm DV. STZ (STZ 3) or vehicle (STZ 0) were ICV-infused by using a 28-gauge Hamilton^®^ (Reno, NV, USA) syringe of 5 μL, and the injection performed via a Harvard Apparatus (Holliston, MA, USA) (model 11 Elite) automated syringe pump at a 0.5 μL/min flowrate. To allow a better diffusion, the needle was left in place for additional 3 min and then, slowly removed. 

### 5.4. Immunization Scheme

The N-terminal tau 12A12 antibody (26-36aa) was produced and characterized by Monoclonal Antibodies Core Facility (MACF) at EMBL-Monterotondo, Rome, Italy (Dott. Alan Sawyer), as previously described [118]. 12A12mAb was purified from hybridoma supernatants according to [1] and its purity was determined using SDS-PAGE and Coomassie staining. In detail, the hybridoma supernatant was precipitated by ammonium sulfate (336 g/L). After precipitation, the solution was centrifuged at 10,000× *g* for 1 h and the pellet was dissolved in PBS and dialyzed against the same buffer. The solution was centrifuged at 10,000× *g* for 30 min and loaded on a HiTrap Protein G HP (GE Healthcare, Chicago, IL, USA) equilibrated with PBS. The column was washed with PBS (5 column volumes). 12A12mAb was eluted with 0.1 M Glycine-HCl, pH 2.7. The fractions containing the antibody were neutralized by 1 M Tris-HCl, pH 9.0, collected and immediately dialyzed against PBS. 12A12mAb concentration was determined by measuring the absorbance at 280 nm. The average yield was 8 mg per liter of cell supernatant. 12A12mAb was ≥95% pure and contained ≤ 1 U/mg of endotoxin (LAL Chromogenic Endotoxin quantitation kit; Thermo Scientific, Waltham, USA, MA).

The dose and route of immunization were based on our prior studies using AD transgenic mice [1,39]. Mice were immunized with 121A12mAb (30 μg/dose) or vehicle-administered (sterile saline). All mice were initially grouped according to their body weight and then, randomized into: (1) mice treated with STZ 0 plus vehicle; (2) mice treated with STZ 0 plus 12A12mAb; (3) mice treated with STZ 3 plus vehicle; (4) mice treated with STZ 3 plus 12A12mAb. Animals received 12A12mAb delivery up to 25 days from STZ infusion, during which they underwent cognitive assessment at two separate intervals, between days 13–18 (short-term immunization regimen, STIR), and between days 20–25 (long-term immunization regimen, LTIR). 12A12mAb immunization was carried out via systemic injections on two alternate days to the lateral vein of the tail, as previously reported [1,39]. In details, mice were placed in a restrainer (Braintree Scientific, Braintree, MA, USA), an inch of the tail was shaved and placed in warm water to dilate the veins. After injection via the lateral tail vein, mice were returned to home cages and kept under general observation. Abnormalities in overall health, home cage nesting, sleeping, feeding, grooming, body weight and condition of the fur of animals were noted. 

Of note, this immunization regimen was previously demonstrated to successfully deliver in vivo a sufficient amount of biologically active (antigen-competent) anti-tau antibody to promote the clearance of the deleterious NH_2_htau peptide accumulating into transgenic AD animals’ hippocampus and to significantly alleviate their behavioral, biochemical, electrophysiological and anatomopathological disease-associated signs [1,39].

### 5.5. Cognitive Assessment: Novel Object Recognition Test (NORT) and Object Place Recognition Task (OPRT)

Hippocampal-dependent, non-associative recognition memory and retention memory of spatial changes were assessed via novel object recognition test (NORT) and object place recognition task (OPRT), respectively. Both OPRT and NORT were performed twice, in two separate time-windows after the ICV-STZ delivery. In both tasks, mice were introduced into an indirectly lit circular open field of 60 cm diameter, closed by a wall of 20 cm high, with a striped card set against the wall as distal cue. The apparatus is made of grey plastic with the floor of the open field divided into several sectors by black lines. Other features of the experimental set-up were described and the NORT and OPRT procedures were performed similarly to our previous investigations [119,120,121]. The cognitive assessment was scheduled to occur at two separate intervals, as follows: (i) 13–14 days after STZ infusion mice were evaluated in the NORT and 17–18 days after STZ infusion the same animals were evaluated in the OPRT; (ii) 20–21 days after STZ infusion mice were evaluated in the NORT and 24–25 days after STZ infusion the same animals were evaluated in the OPRT. The first and the second interval allow us to evaluate the effects of short- and long-term immunization regimens (STIR and LTIR, respectively) on the STZ-induced cognitive impairment (see also the above reported “immunization scheme”).

Either the novel object (i.e., NORT) or the novel position of the familiar object (i.e., OPRT) were changed randomly by alternating left and right position. Time spent in exploration was video-recorded, and exploration defined as the nose pointed within 1 cm range at the object. The percentage of discrimination index (DI) was calculated as time spent into the exploration of the novel object, or novel object location, minus time spent exploring the familiar object/total time of exploration of the two objects (i.e., (time novel object or time novel place − time familiar or time non-displaced object/time novel (or displaced) + time familiar (non-displaced) × 100), as previously reported [1].

In the NORT, we scored the amount of time spent in the active exploration of a new object as compared to the formerly encountered familiar ones, thus gaining an index of spontaneous investigation and development of recognition memory [122,123]. The following experimental procedure was repeated identical after an interval of 7 days (see above) to study the progression of STZ-induced cognitive impairment at two distinct time-windows. Different, in shape and physical features, objects were used during the execution of NORT before and after the 7-day interval. Briefly, mice were familiarized with the environment for a 20-min session, during which the reference level of locomotor activity was gathered. After a 5-min interval, each mouse was positioned back in the arena and allowed to explore for 10 min (training phase) two identical unfamiliar objects placed in the center of the arena. After 24-h delay, mice were placed back into the arena where one of the objects has been replaced by a novel one and allowed to explore for an 8-min session (recognition memory phase). The experimental procedure used for the OPRT was identical to that implemented for NORT, except for performance required consisting in the ability to actively explore a familiar object placed in a novel location, as compared to the familiar location encountered the day before [119,120,121]. As for the NORT, different, in shape and physical features, objects were used during the implementation of OPRT before and after the 7-day interval, as well as different was the spatial place chosen for the novel location of the object.

### 5.6. Tissue Collection, Harvesting and Preparation 

For biochemical analysis, tissue sampling was carried out according to [1]. At the end of period of 12A12mAb administration and two days following the last injection, animals were sacrificed after completion of all the tasks included in the cognitive assessment by cervical dislocation to avoid anesthesia-mediated tau phosphorylation [124]. Brains were collected, the meninges were carefully removed, and dissected hippocampi and cortices were immediately frozen on dry-ice and then, stored at −80 °C until use. 

Frozen hippocampi were diced and homogenized in ice-cold RIPA buffer (50 mM Tris-HCl pH 8, 150 mM NaCl, 1% NP40, 0.1% SDS, 5% sodium deoxycholate) plus proteases inhibitor cocktail (Sigma-Aldrich, P8340) and phosphatase inhibitor cocktail (Sigma-Aldrich, P5726/P2850) for 30 min and centrifuged at 4 °C for 20 min at 13,000 rpm. The amount of total protein was determined by Bradford assay (Protein Assay Dye Reagent Concentrate, Bio-Rad, Hercules, CA, USA).

### 5.7. Western Blot Analysis and Semi-Quantitative Densitometry 

Equal amounts of protein extracts (80–100 μg) were loaded for each blot and in each lane regardless of the antibody used and size-fractionated by SDS-PAGE 7.5–15% linear gradient or Bis-Tris gel 4–12% (NuPage, Invitrogen, Waltham, USA, MA). Samples from different sets of experiments were performed under identical conditions and the average levels of all proteins being studied were roughly comparable between the lysates. After electroblotting onto a 0.2 μm nitrocellulose membrane (Hybond-C Amersham Biosciences, Piscataway, NJ, USA), the filters were blocked in TBS-T containing 5% non-fat dried milk for 1 hr at room temperature or overnight at 4 °C and then, incubated with appropriate primary antisera/antibodies diluted in TBS overnight at 4 °C. After 1 h incubation with secondary anti-mouse or anti-rabbit anti-IgG immunoglobulins conjugated with horseradish peroxidase, the blots were developed by using the enhanced chemiluminescence Western blotting immunodetection system (ECL) (Amersham, Arlington Heights, IL, USA). The signal detection was performed by using the iBright Imaging Systems (ThermoFisher Scientific, Waltham, MA, USA). Semi-quantitative densitometry of the intensity signals of bands was carried out following normalization with β-actin level, used as loading control for every target protein and for every experiment performed. Representative blot against β-actin was shown in final figures. Final figures were assembled by using Adobe Illustrator 10 and Adobe Photoshop 6 and quantitative analysis of acquired images was performed by using ImageJ 1.46 (http://imagej.nih.gov/ij/, accessed on 1 October 2012). 

### 5.8. Oxidative Stress and Mitochondrial Analysis

(a) Tissue homogenate preparation

For mitochondrial analyses, brain cortices from the four experimental groups were stored at –80 °C until assayed. The PBI-Shredder—an auxiliary high-resolution respirometry (HRR) Tool—was used to prepare homogenate of frozen tissue specimens in 0.2 M phosphate buffer (pH 8.0) according to [39,74] with high reproducibility of mitochondrial function evaluated with HRR by means of Oxygraph-2 k OROBOROS^®^ (Innsbruck, Austria). Homogenate protein content was determined as in [125].

(b) Assessment of Cellular and Mitochondrial ROS Production

Superoxide anion radical (O2^−•^) was detected both according to the adrenochrome method and by using the MitoSOX dye [126] in order to distinguish the specific mitochondrial ROS production from that occurring by other sources. In the adrenochrome method, the increase of absorbance at 480 nm (i.e., the conversion of epinephrine (no color) into adrenochrome (pink) with 1:1 stoichiometry) is the result of superoxide formation and was obtained by adding 0.5 mg protein homogenate to 2 mL of PBS in the presence of epinephrine (1 mM). Absorbance increase was measured using a PerkinElmer lambda-5 spectrophotometer equipped with a thermostated holder. 

To specifically detect O2^−•^ production of mitochondrial origin, use was made of MitoSOX Red, a specific mitochondrial dye which is highly and exclusively sensitive to superoxide [126,127] but not to other reactive oxygen/nitrogen species. MitoSOX Red is selectively targeted to mitochondria where it accumulates as a function of mitochondrial membrane potential and exhibits fluorescence upon oxidation by superoxide and subsequent binding to mitochondrial DNA. The reliability of the method is strictly dependent on the fact that cells homogenate containing respiring mitochondria. Brain cortex homogenate (0.1 mg protein/mL) in PBS was pre-incubated with succinate (10 mM) plus ADP (2.5 mM) for 2 min at 25 °C, which provide a necessary energy source for the success of the procedure. After addition of 1 μM MitoSOX red, either in the absence or presence of different compounds, followed by 15 min incubation, fluorescence emission at 580 nm was measured using a Perkin-Elmer LS-50B Luminescence Spectrofluorometer (Perkin-Elmer Applied Biosystems, Foster City, CA, USA). 

(c) Lipid peroxidation assays

The membrane peroxidation was measured by means of the sensitive technique of cis-parinaric acid (PnAc) fluorescence loss, as reported in [64,128]. Homogenate (0.5 mg of protein in 4 mL PBS) was pre-incubated with 5 μM PnAc (Molecular Probes, Eugene, OR) for 15 min in the dark to allow its integration in membranes. Afterward, the suspension was centrifuged, and the pellet was re-suspended in 2.0 mL of PBS. The fluorescence (318 nm excitation and 410 nm emission) was measured every 3 min for 30 min in a Perkin Elmer LS-50B Luminescence Spectrofluorometer equipped with a thermostated cuvette at 25 °C and a stirring device. No PnAc fluorescence loss (i.e., no lipid peroxidation) was detected when the probe was added to medium but without homogenate. Loss of PnAc fluorescence was used as an index of lipid peroxidation and, accordingly, the greater lipid peroxidation, the less fluorescence was detected.

(d) Measurement of COX activity 

Cytochrome c oxidase (COX) activity was measured by spectrophotometric standard method [74]. Assay was performed at least in triplicate by using homogenate tissues, which were subjected to three freeze–thaw cycles in order to disrupt membranes and expose mitochondrial enzyme.

(e) Determination of GSH level

GSH content was estimated by using a thiol specific reagent, 5,5′-dithiobis-(2-nitrobenzoic acid) (DTNB) [68,129]. Briefly, the spectrophotometric assay method involves oxidation of GSH by DTNB to form the yellow derivative 5′-thio-2-nitrobenzoic acid (TNB), which is measurable at 412 nm. The glutathione disulfide (GSSG) formed, as well as the GSSG present in the sample, can be recycled to GSH by glutathione reductase in the presence of NADPH. Both the total amount of GSH and of GSSG in the samples were calculated from a standard curve obtained by plotting known amounts of either GSH or GSSG versus the rate of change of absorbance.

(f) NADPH and NADH level measurements

Nicotinamide adenine dinucleotide levels were performed by using the method involving 3-(4,5-dimethylthiazolyl-2)-2,5-diphenyltetrazolium bromide (MTT), as terminal electron acceptor, with phenazine ethosulfate (PES), as an electron carrier, according to [127]. The reduction rate of the MTT is proportional to the concentration of the nicotinamide adenine dinucleotide and, thus, cycling time must be strictly kept the same for each assay. For NADH determination, the first step is catalyzed by alcohol dehydrogenase (ADH) in the presence of ethanol and the rate of reduction of MTT is proportional to the concentration of the coenzyme. In the procedure for the determination of NADP(H), ethanol and ADH are replaced by glucose-6-phosphate and glucose-6-phosphate dehydrogenase (for details see [130]). 

(g) Measurement of mitochondrial RC Complex I–V activities

Complex I–V enzymatic activities were assayed photometrically at 25 °C, as in [39]. Each assay was performed at least in triplicate by using tissue homogenates subjected to three freeze–thaw cycles to disrupt membranes and expose enzymes. Homogenate from each tissue sample was suspended in 0.3 mL of the respiration medium (consisting of 210 mM mannitol, 70 mM sucrose, 20 mM Tris/HCl, 5 mM KH_2_PO_4_/K2HPO_4_, (pH 7.4), 3 mM MgCl_2_) and subdivided to perform three assays, essentially as described previously [131], which rely on the sequential addition of reagents to measure the activities of: (i) NADH:ubiquinone oxidoreductase (complex I) followed by ATP synthase (complex V); (ii) succinate:ubiquinone oxidoreductase (complex II); (iii) cytochrome c oxidase (complex IV) followed by cytochrome c oxidoreductase (complex III).

COX activity was also determined in tissue homogenate by using ascorbate (ASC) plus tetramethyl *p*-phenylenediamine (TMPD) as an electron donor system [132] and by measuring O_2_ consumption polarographically.

(h) Measurement of ATP levels 

Tissue homogenates were subjected to perchloric acid extraction as described in [133]. Briefly, tissues were homogenized in 600 μL of pre-cooled 10% perchloric acid and then centrifuged at 14,000× *g* for 10 min, 4 °C. The amount of tissue ATP was determined enzymatically in KOH neutralized extracts, as described in [39].

### 5.9. Immunofluorescence and Fluorescent Acquisition 

Animals were intracardially perfused with ice-cold phosphate-buffered-saline (PBS) 0.1 mol/liter pH7.4 using a 30 mL syringe to remove blood contamination and then, with 4% paraformaldehyde (PFA) solution in PBS. After that, brains were carefully removed from the skull and post-fixed in 4% PFA solution for 16 h overnight at + 4 °C and then, passed into 30% sucrose solution in 0.1 mol/liter phosphate buffer for 48–72 h until equilibration. The brains were frozen by immersion in ice-cold isopentane for 3 min before being sealed into vials and stored at −80 °C until use.

Frozen sections (10 µm thickness) were obtained by a Laica cryostat and placed on BDH slides (Milan, Italy). After three washes in PBS, the sections were exposed to antigen retrieval (0.25% trypsin in PBS solution, 7 min) and blocking/permeabilizing (5% BSA, 10% NGS and 0.5% Triton X 100 in PBS, 6 h) steps, all at room temperature and probed overnight at 4 °C with primary antibodies (anti-synapsin I antibody rabbit AB1543P Millipore, anti-α-synuclein rabbit (D37A6) XP Cell Signaling)) diluted 1:100 in PBS buffer containing 2.5% BSA, 5 % NGS and 0.3% Triton X 100. After three washes in PBS 1X with 0.1% Triton X 100, the sections were incubated for 2 h at room temperature with the secondary antibody (Cy3, red, 1:300, goat; Cy2, green, 1:300 Jackson ImmunoResearch, Europe Ltd., Sufolk, UK) diluted 1:300 in PBS buffer containing 1.25% BSA, 2.5% NGS and 0.15% Triton X 100. After three washes in PBS, nuclear counterstaining was performed by using fluoroshield with DAPI (F6057, Sigma-Aldrich, St. Louis, MO, USA). Images (20×) are representative of at least three independent experiments and were acquired with spinning disk system for fast fluorescence confocal microscopy, with led or laser light source—Crest Optics, (Crisel Instruments, Rome, Italy). Olympus Confocal Microscope Quantitative image analysis was performed by using Metamorph Research Imaging and ImageJ 1.4 software1.

Paraffine-included sections (7 µm thickness) were produced and placed on BDH slides (Milan, Italy), air-dried and stored at −20 °C. Dewaxed sections were exposed to quenching (50 mM NH_4_Cl, 5 min), antigen retrieval (0.05% trypsin-EDTA solution, 2 min) and blocking/permeabilizing (1% BSA and 0.5% Triton X 100 in PBS, 30 min) steps, and probed overnight with primary antibody (Caspase-cleaved protein (CCP, NH_2_-tau antiserum (D_25_-(QGGYTMHQDQ) epitope, phosphorylation-independent state diluted 1:200) in PBS (10 mM phosphate buffer and 150 mM NaCl; pH 7.5) [39]. The secondary antibody was Cy2 (green) conjugated species-specific antibody (1:1000; donkey; Jackson ImmunoResearch, Europe Ltd., Suffolk, UK) diluted in PBS. Washing steps were performed in PBS containing 0.05% Tween 20. Nuclear counterstaining was performed with 1 μM DAPI solution (D9542; Sigma-Aldrich, St. Louis, MO, USA). Negative control (isotype) was carried out in parallel with the omission of primary antibody and used for appropriate background subtractions. Serial images were analyzed, and selected images were digitally acquired (8-tiff) by NIS software connected to fluorescent direct microscope (Eclipse Ni; Nikon, Tokyo, Japan). 

### 5.10. Histopathological Analysis

For histopathological analysis of hippocampus, animals were intracardially perfused with ice-cold phosphate-buffered-saline (PBS) using a 30 mL syringe to remove blood contamination, the intact brain was isolated, cleaned with PBS with the utmost caution so as not to inflict damage. Brain tissues were dipped in tubes containing 10% formalin solution for the purpose of fixation. Tubes were left over at room temperature for 24 h. Later, brain tissues were shifted to melted paraffin wax and solidified. Several sections of the tissues of 7 μm thickness were manually trimmed using a microtome (HM325 rotary microtome; Microm, Rijswijk, Netherland). The tissue slices were subsequently dewaxed, followed by dehydration with increased gradient concentrations of an aqueous-alcohol solution. The slices were stained with hematoxylin and eosin dye, placed on glass slides and observed under a light microscope. The viable and dead neuronal cells were identified and analyzed.

Fid5.11. Data management and statistical analysis

Data were expressed as means ± standard error of the mean (S.E.M.). All data were representative of at least three separate experiments (n = independent experiments). For behavioral, 9 mice were used per experiment per condition. For Western blotting and for oxidative stress and mitochondrial analyses, 11 mice were used per experiment per condition. For immunofluorescence and histology, 3 mice were used per experiment per condition. Statistically significant differences were analyzed by one-way or two-way analysis of variance (ANOVA) followed by Bonferroni’s post-hoc test for multiple comparison among more than two groups. *p* < 0.05 was accepted as statistically significant (* *p* < 0.05; ** *p* < 0.01; *** *p* < 0.0005; **** *p* < 0.0001). All statistical analyses were performed using GraphPad Prism 8 software.

## Data Availability

All the data used and/or analyzed for the current study is contained in the article. All other datasets are available from the corresponding author upon reasonable request.

## Supplementary Materials

The following are available online at https://www.mdpi.com/article/10.3390/ijms222212158/s1.

## Abbreviations

Alzheimer’s Disease (AD); Familial Alzheimer’s Disease (fAD); Sporadic Alzheimer’s Disease (sAD); Amyloid beta (Aβ); streptozotocin (STZ); Intra-cerebroventricular (ICV); monoclonal Antibody (mAb); short-term immunization regimen (STIR); long-term immunization regimen (LTIR); amyloid precursor protein (APP); β-Secretase 1 (BACE-1); presenilin 1 (PS1); glial fibrillary acidic protein (GFAP); ionized calcium-binding adapter molecule 1 (Iba-1); insulin-like growth factor (IGF); insulin receptor (IR); phosphatidylinositol 3-kinase (PI3K); protein kinase B (AKT); glycogen synthase kinase 3 beta (GSK-3β); 5′ AMP-activated protein kinase (AMPK); novel object recognition test (NORT); object place recognition test (OPRT); cytochrome c oxidase (COX); hematoxylin and eosin (H&E); Food and Drug Administration (FDA); familial object (FO); novel object (NO); familial place (FP); novel place (NP).
