# Supplementary material for: Tau Cleavage Contributes to Cognitive Dysfunction in Strepto-Zotocin-Induced Sporadic Alzheimer’s Disease (sAD) Mouse Model
Source: Int J Mol Sci. 2021 Nov 10;22(22):12158. doi: 10.3390/ijms222212158 (PMC8618605; doi:10.3390/ijms222212158)
Supplement: Supplementary file 1 [file ijms-22-12158-s001.zip › ijms-1391018-supplementary.pdf]

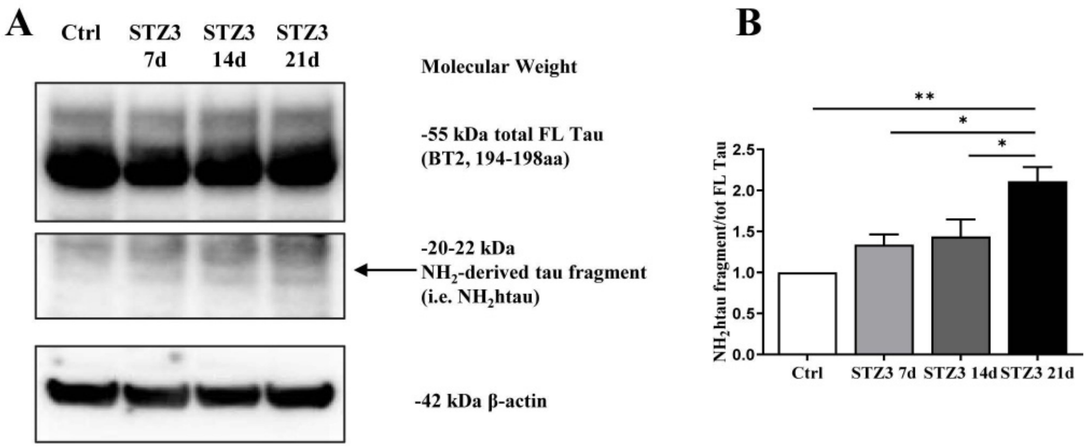

Figure S1 . The neurotoxic 20-22kDa NH_2htau peptide gradually accumulates into animals' hippocampus following continuous STZ injection. (A) Hippocampal protein homogenates from naïve untreated mice (Ctrl) and mice injected with 3mg/Kg STZ (STZ 3) for 7, 14, 21 days were analyzed by SDS-PAGE Western blotting with BT2, the pan-tau antibody directed against the 194-198 amino acids of full-length protein, to detect the steady-state expression level of the neurotoxic 20-22kDa NH_2htau peptide over time. (B) Semi-quantitative densitometry of the intensity signals of bands was carried out following normalization with β-actin level used as loading control. Values are from at least three independent experiments and statistically significant differences were calculated by one-way ANOVA followed by Bonferroni's post-hoc test for multiple comparison among more than two groups. $p < 0.05$ was accepted as statistically significant (*$p < 0.05$; **$p < 0.01$).
